# Discrimination of Cultivated Regions of Soybeans (*Glycine max*) Based on Multivariate Data Analysis of Volatile Metabolite Profiles

**DOI:** 10.3390/molecules25030763

**Published:** 2020-02-10

**Authors:** So-Yeon Kim, So Young Kim, Sang Mi Lee, Do Yup Lee, Byeung Kon Shin, Dong Jin Kang, Hyung-Kyoon Choi, Young-Suk Kim

**Affiliations:** 1 Department of Food Science and Engineering, Ewha Womans University, Seoul 03760, Korea; soyeon3143@daum.net (S.-Y.K.); 0303soyoung@naver.com (S.Y.K.); smlee78@ewha.ac.kr (S.M.L.); 2 Department of Agricultural Biotechnology, Research Institute for Agricultural and Life Sciences, Seoul National University, Seoul 08779, Korea; rome73@snu.ac.kr; 3 National Agricultural Products Quality Management Service, Gimcheon 39660, Korea; sbkon1@korea.kr (B.K.S.); kdj1216@korea.kr (D.J.K.); 4 College of Pharmacy, Chung-Ang University, Seoul 06974, Korea

**Keywords:** gas chromatography-mass spectrometry, solid-phase microextraction, soybean, origin discrimination, volatile compounds

## Abstract

Soybean (*Glycine max*) is a major crop cultivated in various regions and consumed globally. The formation of volatile compounds in soybeans is influenced by the cultivar as well as environmental factors, such as the climate and soil in the cultivation areas. This study used gas chromatography-mass spectrometry (GC-MS) combined by headspace solid-phase microextraction (HS-SPME) to analyze the volatile compounds of soybeans cultivated in Korea, China, and North America. The multivariate data analysis of partial least square-discriminant analysis (PLS-DA), and hierarchical clustering analysis (HCA) were then applied to GC-MS data sets. The soybeans could be clearly discriminated according to their geographical origins on the PLS-DA score plot. In particular, 25 volatile compounds, including terpenes (limonene, myrcene), esters (ethyl hexanoate, butyl butanoate, butyl prop-2-enoate, butyl acetate, butyl propanoate), aldehydes (nonanal, heptanal, *(E)*-hex-2-enal, *(E)*-hept-2-enal, acetaldehyde) were main contributors to the discrimination of soybeans cultivated in China from those cultivated in other regions in the PLS-DA score plot. On the other hand, 15 volatile compounds, such as 2-ethylhexan-1-ol, 2,5-dimethylhexan-2-ol, octanal, and heptanal, were related to Korean soybeans located on the negative PLS 2 axis, whereas 12 volatile compounds, such as oct-1-en-3-ol, heptan-4-ol, butyl butanoate, and butyl acetate, were responsible for North American soybeans. However, the multivariate statistical analysis (PLS-DA) was not able to clearly distinguish soybeans cultivated in Korea, except for those from the Gyeonggi and Kyeongsangbuk provinces.

## 1. Introduction

Soybean (*Glycine max*) is among the most important crops in the world and is extensively used in the production of soybean flour, soybean milk, fermented products, and oil for consumption by both humans and animals, mainly due to its high protein and fat contents [1]. It is generally accepted that soybean cultivation originated in China, but nowadays, soybeans are produced worldwide, including in North America, South America, and Asia [1]. The importing and exporting of agricultural products are increasing globally due to the expansion of the free-trade agreements. These circumstances have resulted in some foreign soybeans with unclear origins being distributed as domestic ones in Korea, which can lead to consumer distrust about the market [2]. The National Agricultural Products Quality Management Service in Korea introduced an agricultural food origin labeling system in 1991 to protect domestic agricultural producers and consumers [2]. Soybeans have been included in this system since 2017, and traders must now mark the origins of all products advertised for sale [2]. 

The properties and qualities of soybeans can be significantly affected by their cultivation region because each production area has different growing conditions, such as temperature, precipitation, and soil characteristics [3,4,5]. In particular, some previous studies have demonstrated that light and water characteristics and growth temperatures significantly affect the formation of volatile metabolites, such as alcohol and aldehydes in plants [6,7]. In addition, Grieshop et al. and Cherry et al. demonstrated that the environmental growing conditions of soybeans could change the synthesis pathways of proteins and fats, thereby affecting their chemical compositions [8,9].

Volatile components of soybeans have been found to be mainly derived from carbohydrates, proteins, and lipids via enzymatic reactions, autoxidation, and/or other chemical reactions during both storage and cultivation [10]. Lee et al. explained that major aroma constituents of soybean include hexanal, 1-octen-3-ol, γ-butyrolactone, maltol, and phenylethyl alcohol [11]. Also, Boué et al. and Dings et al. identified hexan-1-ol, octan-3-one, oct-1-en-3-ol, ethanol, octanal, 2-propanone, hexan-1-al, and 1-pentan-3-ol—most of which could be lipid oxidative degradation products—as major volatiles in soybean using Tenax trapping and solid-phase microextraction (SPME) combined by gas chromatography-mass spectrometry (GC-MS) analysis [12,13]. Since the composition of volatile compounds in soybeans can vary depending on their cultivation conditions, it might be feasible to use data sets of volatile components to distinguish where soybeans originate from.

Metabolomics is a commonly used tool for the identification and quantification of whole metabolites in biological samples [14]. Metabolite profiling has been performed using various instrumental methods, including mass spectrometry (MS) and nuclear magnetic resonance (NMR) spectroscopy [15,16,17]. These approaches have been successfully used to determine the geographical origins of various agricultural products [18,19,20]. Metabolomics approaches based on several different types of instrumental methods have been recently used to distinguish soybeans of different geographical origins [21,22]. Liquid-chromatography-orbitrap mass spectrometry (LC-Orbitrap MS) and gas-chromatography time-of-flight mass chromatography (GC-TOF MS) have been used to obtain the metabolic fingerprints of soybeans cultivated domestically in different provinces of Korea [21]. Fourier-transform infrared (FT-IR) spectroscopy has also been combined with multivariate statistical analysis to distinguish the geographical origins of Chinese and Korean soybeans [22]. However, while GC-MS has been widely used to discriminate the origins of foodstuffs and agricultural products, such as green tea, omija fruit, and honey, mainly due to its high resolution and sensitivity, in particular, in the analysis of volatile compounds, this method has not previously been applied to distinguish soybeans according to their cultivation regions based on the data sets of volatiles [14,23,24,25]. Therefore, we aimed to determine the feasibility of discriminating soybeans according to the cultivation regions using a GC-MS-based metabolomics approach in this study.

## 2. Results and Discussion

### 2.1. Profiling of Total Volatile Compounds in Soybeans

In total, 146 volatile compounds were identified in GC-MS data sets obtained from soybean samples of different geographical origins. Tables S1–S3 indicate that diverse lipid-derived volatile compounds and terpenes were detected in this study. Previous studies have found the major volatiles of soybeans to be ethanol, 1-octen-3-ol, maltol, phenylethyl alcohol, hexanal, octanal, 2-propanone, and γ-butyrolactone [11,12]. All of these volatile compounds were detected in the present study with the exception of maltol, which could have been due to the use of different extraction techniques [11]—the present study employed headspace extraction using SPME, which generally focuses on the detection of highly volatile compounds with low boiling points.

The 84 volatile compounds detected in the soybeans cultivated in Korea comprised of 1 acid, 23 alcohols, 9 aldehydes, 4 esters, 6 furans, 6 benzenes, 10 ketones, 3 lactones, 3 nitrogen-containing compounds, 2 sulfur-containing compounds, 10 hydrocarbons, 6 terpenes, and 1 phenol. Certain alcohols, such as 2-ethylhexan-1-ol, predominated, followed by ketones, such as propan-2-one, while terpenes were found at low levels in most samples. Unlike soybeans grown in China and North America, pyrazines were not identified in those cultivated in Korea.

The 124 volatile compounds identified in the soybeans cultivated in China comprised of 2 acids, 25 alcohols, 13 aldehydes, 13 esters, 4 furans, 8 benzenes, 17 ketones, 4 lactones, 3 nitrogen-containing compounds, 2 sulfur-containing compounds, 16 hydrocarbons, 11 terpenes, 3 phenols, and 3 pyrazines. Among them, 3-methylheptan-4-one was detected at higher levels compared to those cultivated in Korea and North America, and there was a greater diversity of terpenes in the Chinese soybeans.

The soybeans cultivated in North America contained 50 volatile compounds: 1 acid, 16 alcohols, 5 aldehydes, 4 esters, 2 furans, 4 benzenes, 4 ketones, 3 lactones, 1 nitrogen-containing compound, 1 sulfur-containing compound, 5 hydrocarbons, 2 terpenes, 1 phenol, and 1 pyrazine. The number of volatile compounds detected in North American soybeans was clearly smaller than in those from other cultivation areas, but there was a greater diversity of alcohol. Oct-1-en-3-ol was detected at higher levels, while propan-2-one and 2-methylprop-1-ene were present at lower levels in North American soybeans. The only pyrazine detected was 2-methylpyrazine. Among esters, the content of 3-hydroxy-2,4,4-trimethylpentyl 2-methylpropanoate was higher in soybeans from Indiana province (IN) than in those of other regions of North America.

Several enzymes of soybeans have been studied by various researchers, including lipoxygenase, lipase, urease, amylase, and protease [26,27]. In particular, soybeans are known to be a rich source of lipoxygenase [27], which is one of several enzymes used to produce aldehydes and alcohols via enzymatic oxidation [28]. This study found hexanal (13-linoleate hydroperoxide) and heptanal (11-linoleate hydroperoxide)—known as the major oxidative products from linoleate hydroperoxides— in most of the cultivation regions, as were octanal (11-oleate hydroperoxide) and nonanal (9-/10-oleate hydroperoxide) [29], which are known to be decomposition products of oleate hydroperoxides [30].

Benelli et al. found that the amount of hexanal was related to precipitation and light conditions in the cultivation area [7]. Table 1 [31] presents the differences in precipitation between the cultivation regions, whereas the amount of hexanal did not differ significantly between the geographical regions studied. In this study, alcohols—which are known to be secondary oxidative products of unsaturated fatty acids [29]—predominated in soybeans from Korea, China, and North America, among which pentan-1-ol and hexan-1-ol (both are derived from 13-linoleate hydroperoxide [30,32]) were observed in most samples. As mentioned above, oct-1-en-3-ol (produced from 10-linoleate hydroperoxide [32]) was the most abundant alcohol in soybeans cultivated in North America. On the other hand, furans can be produced from the oxidation of polyunsaturated fatty acids and carotenoids [33], and 2-alkylfurans are commonly derived from lipid degradation [34]. 2-Methylfuran, 2-ethylfuran, and 2-pentylfuran were detected in soybeans from Korea and China, whereas 2-methylfuran was not found in soybeans from North America.

Several ketones were also identified in soybeans from Korea, China, and North America. Other diverse ketones that are mainly formed from unsaturated fatty acids (e.g., linoleic acid) by lipoxygenase were found in soybeans from China [35,36]. Certain ketones, such as propan-2-one, butan-2-one, and 3-methylheptane-4-one, were commonly found in samples from China. Cheesbroug et al. and Gulen et al. explained that the activities of enzymes, such as peroxidase, increased with the temperature at which the plants were grown [37,38]. Also, some previous studies have reported that lipoxygenase activity is affected by the minimum mean temperature from flowering to maturity [39], which affects the formation of volatile compounds [40]. Table 1 indicates that the annual mean temperature was higher in China (excluding the northeast region) than in other cultivation areas (Korea and North America). It could, therefore, be assumed that the formation of various ketones in soybeans from China is due to high lipoxygenase activity related to the temperatures of their cultivation areas.

Diverse terpenes that occur naturally as metabolites are commonly found in plants [41]. In general, terpenes are produced from isopentenyl diphosphate, which is elongated to geranyl diphosphate, farnesyl diphosphate, and geranylgeranyl diphosphate [42]. Those terpenes were identified in all of the present cultivated areas but showed the greatest abundance and variety in China. The 11 terpenes of α-pinene, α-thujene, sabinene, l-phellandrene, myrcene, α-terpinene, limonene, β-phellandrene, γ-terpinene, terpinolene, and α-cedrene were detected in soybeans from China. The formation of terpenes could depend on various factors, such as cultivar and region [43]. Marais reported that certain factors, such as increased temperature and acidic conditions, could affect the concentration and diversity of terpenes formed [43]. Also, terpene synthases could be affected by CO_2_ levels [40]. According to Planbureau voor de Leefomgeving (PBL) Netherland Environmental Assessment Agency, China showed the largest CO_2_ emissions in 2016 [44]. In particular, limonene derived from geranyl pyrophosphate was identified in all samples from China. A previous study suggested that a higher CO_2_ concentration could enhance the activity of limonene synthase [40]. Therefore, the formation of limonene could be significantly affected by CO_2_ concentration as well as other factors, such as temperature.

### 2.2. Discrimination of Soybeans by Different Geographical Origins

In order to discriminate soybeans according to their geographical origins, the relationship between soybeans from different cultivation regions and their volatile profiles was investigated. GC-MS data sets were processed using unsupervised statistical analysis (principal components analysis (PCA) and hierarchical clustering analysis (HCA)) as well as supervised statistical analysis (partial least square-discriminant analysis (PLS-DA)) [45]. PCA, HCA, and PLS-DA were performed to identify the differences in volatiles profiles obtained from GC-MS analyses of soybeans of different geographical origins.

**Figure 1:**
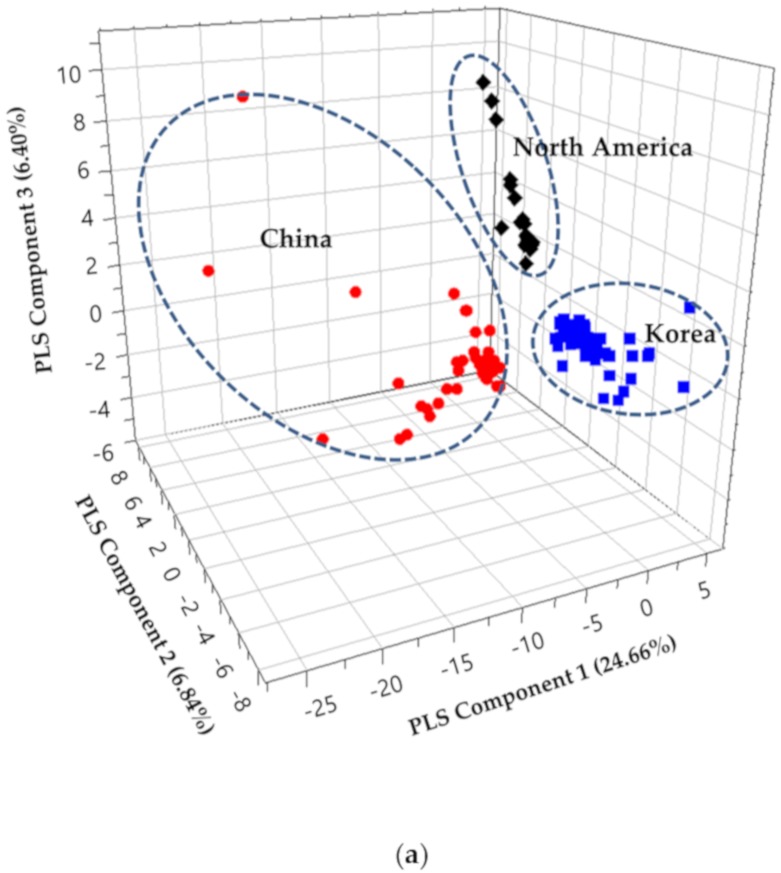

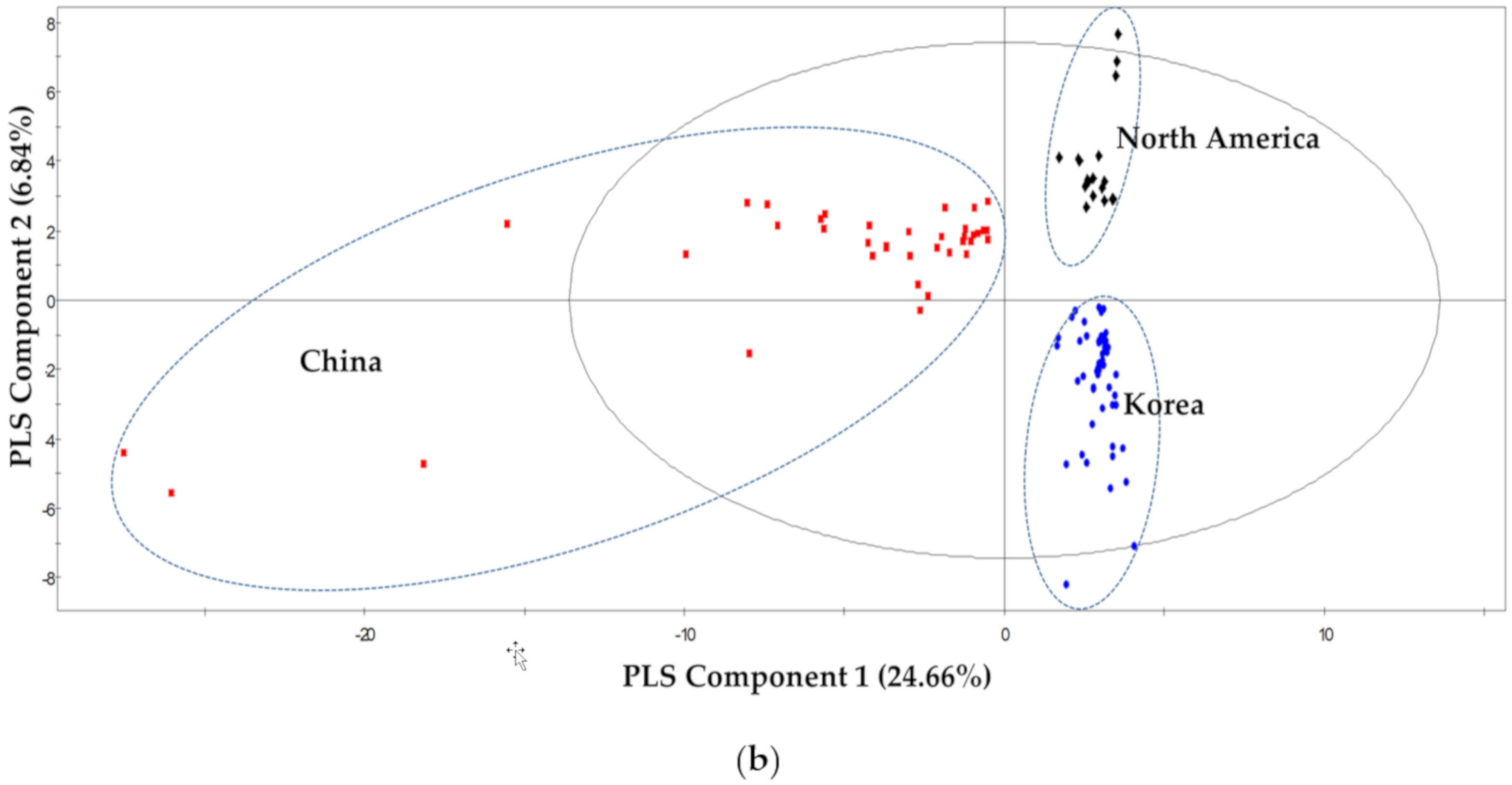
Partial least square-discriminant analysis (PLS-DA) score plot of soybean samples from different cultivation areas; (**a**) 3D score plot; (**b**) score plot PLS[1]-PLS[2], indicating the separation between different cultivation areas.

The results of PCA were distinguished by their geographical origins (data not shown). Since both results of PCA and PLS-DA on score plots were similar, only PLS-DA results were presented to show the separation of samples according to the cultivation area (Figure 1). In addition, partial least square (PLS) components 1, 2, and 3 in the PLS PLS-DA 3D score plot for soybeans of different origins together explained 37.9% of the total variance: 24.66%, 6.84%, and 6.40%, respectively (Figure 1a). The PLS-DA score plot for PLS component 1 and PLS component 2 is presented (Figure 1b). The parameters of the cross-validation modeling were component 3, with R^2^X = 0.379, R^2^Y = 0.788, and Q^2^(cum) = 0.709. After 100 times permutations, R^2^ = 0.177 and Q^2^ = −0.219 were obtained.

Some previous studies have shown that the chemical compositions of soybeans can vary significantly with differences in soils, fertilizer treatment, and climatic conditions, as well as other environmental factors [46,47,48]. Grieshop and Fashey showed that soybeans from China had greater crude protein content than those from North America [8]. Also, Shi et al. [47] demonstrated that soybeans from Korea contained more protein and less oil than those from North America. On the other hand, soybeans from China have been shown to have lower lipid concentration than those from North America [9]. Volatile compounds of soybeans are produced by nonvolatile precursors, such as lipids, sugars, and proteins [49]. In particular, oxidative degradation of lipids can lead to the formation of diverse volatiles. Certain lipid-derived compounds, such as oct-1-en-3-ol, differed significantly between soybeans from North America and those cultivated in other regions, which could be due to the higher lipid concentration of North American soybeans. On the other hand, the amounts of benzaldehyde, 2,6-dimethylpyrazine, and 2,5-dimethylpyrazine, which are known to be mainly produced by amino acids as major precursors [50], differed significantly between soybeans from China and those cultivated in other regions. This could be at least partially due to the differences in protein content between soybeans from different cultivation regions [46,48]. However, their exact formation mechanisms remain unclear, and they could involve both biological and chemical mechanisms during the cultivation and storage of the soybeans.

Medic et al. reported that the constituents of soybeans could be significantly altered by diverse environmental factors exerting complex combined effects [50]. This situation makes it difficult to explain how specific environmental factors influence the formation of volatile components in soybeans. As shown in Figure 1, the soybean samples could be divided into three groups for Korea, China, and North America. Soybeans from China are located in the area of negatively-related PLS component 1 in this score plot, whereas those from Korea and North America are located in the positions of both positively-related PLS component 1. Soybeans from North America are located in the positions of positively-related PLS component 2 in this score plot, whereas those from Korea are located in the positions of negatively-related PLS component 2.

**Figure 2:**
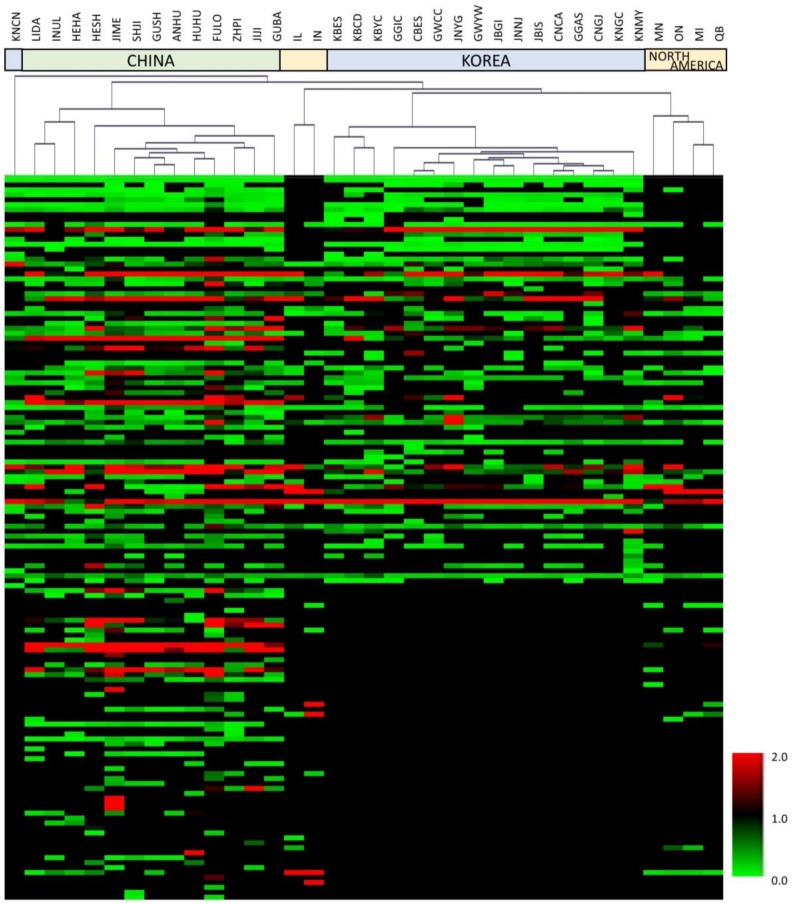
Heatmap generated by a hierarchical clustering analysis of 146 metabolites.

Table 2 and Table 3 list the main volatile metabolites identified according to the variable importance plot (VIP) values of >1.20. A VIP value >1 suggests that a compound plays a predominant role in the separation of groups [51]. The major volatile metabolites contributing to the positive PLS 1 axis were 2-ethylhexan-1-ol, while those in the negative axis of PLS component 1 were heptan-4-ol, butan-1-ol, butyl butanoate, octanal, butyl prop-2-enoate, 5-methyl-2-propan-2-ylcyclohexan-1-ol, butyl acetate, butyl propanoate, nonanal, toluene, heptanel, heptan-4-one, 5-ethyloxolan-2-one, 1,2,3-trimethylbenzene, heptan-2-one, (*E*)-hex-2-enal, ethyl hexanoate, (*E*)-hept-2-enal, limonene, 1-butoxybutane, 2-pentylfuran, acetaldehyde, myrcene, and 3-hydroxybutan-2-one, whereas those in the negative axis of PLS component 1 were found in all soybeans from China. On the other hand, the main volatile metabolites that contribute to the negative PLS 2 axis were 2-ethylhexan-1-ol, 2,5-dimethylhexan-2-ol, styrene, 2-methylfuran, 2- methylprop-2-ene, propan-2-one, 2-methylprop-2-enal, hexane, methyl acetate, 2-methylpentan-1-ol, octanal, butyl prop-2-enoate, 1-methyoxypropan-2-ol, heptanal, and toluene, whereas those in the positive PLS 2 axis were oct-1-en-3-ol, nonane, 4-methyloxolan-2-one, heptan-4-ol, butan-1-ol, octan-3-one, butyl butanoate, 3-hydroxy-2,4,4-trimethylpentyl 2-methylpropanoate, 5-methyl-2-propan-2-ylcyclohexan-1-ol, butyl acetate, butyl propanoate, and nonanal. In Figure 2, soybeans from each country are clustered according to their cultivation regions. The figure shows that soybeans from Korea were clustered more closely than the others, which is possibly due to the much smaller land area of that country (100,339 km^2^) compared to China (9,596,951 km^2^), Canada (9,984,670 km^2^), and North America (9,826,676 km^2^).

Figure 2 shows the HCA dendrogram with its associated heatmap in which all of the samples are grouped in terms of their nearness or similarity [52]. The figure shows that all of the samples could be clustered into two groups except for Kyeongsangnam province Changnyeong (KNCN): group I consisted of 13 soybean samples cultivated in China, and group II comprised of 22 soybean samples from Korea and North America. The amounts of terpenes and esters were greater in group I than in group II. In group II, soybean samples from Korea—except for Kyeongsangnam province Changnyeong (KNCN)—and North America were classified into the subgroup. Among soybean samples grown in North America, those from Illinois (IL) and Indiana (IN) provinces could be distinguished from the others. Table S3 indicates that the samples from Illinois and Indiana provinces were found to contain greater amounts of alcohol than other North American soybeans (samples MI, MN, ON, and QB). The annual mean precipitations were similar across North America, but the annual mean temperatures were higher in Illinois and Indiana than in the other regions. Wills et al. reported that the concentration of esters and alcohols was positively related to temperature [53]. Therefore, it could be inferred that the formation of volatile compounds was affected by the cultivation temperature in soybeans from North America.

**Figure 3:**
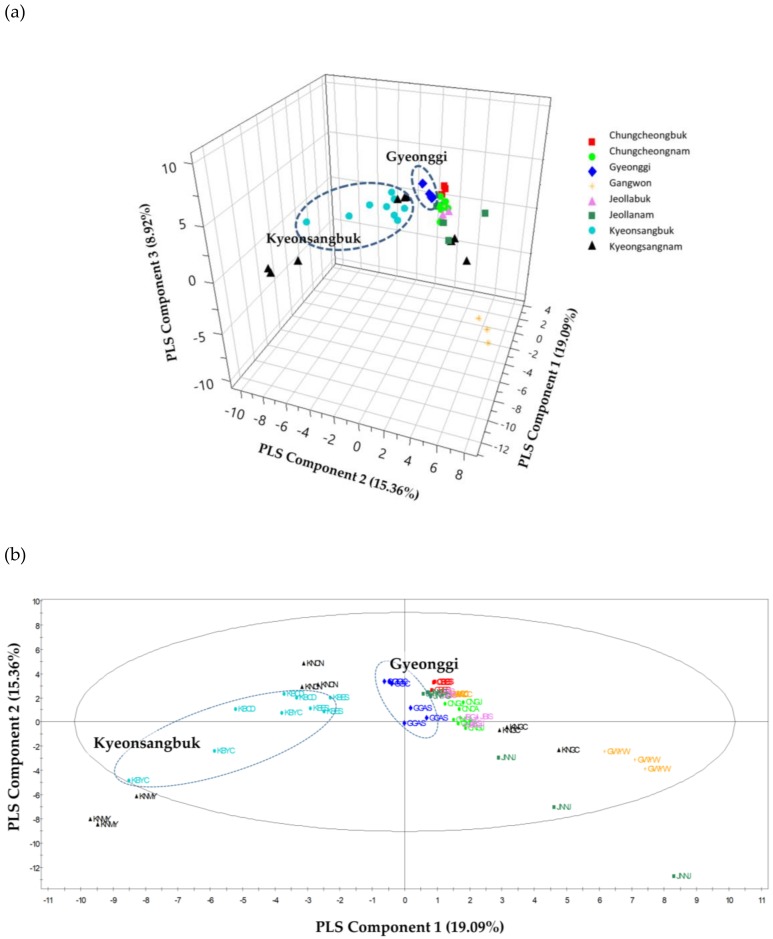
PLS-DA score plot of soybeans from Korea on the basis of volatile metabolites: (**a**) 3D score plot; (**b**) score plot PLS[1]-PLS[2]. GGIC—Gyeonggi province Anseong, GGAS—Gyeonggi province Icheon, GWCC—Gangwon province Chuncheon, GWYW—Gangwon province Yeongwol, CBES—Chungcheongbuk province Eumseong, CNCA— Chungcheongnam province Cheonan, CNGJ—Chungcheongnam province Gongju, JBGJ—Jeollabuk province Gimje, JBIS—Jeollabuk province Imsil, JNNJ—Jeollanam province Naju, JNYG—Jeollanam province Yeonggwang, KBCD—Kyeongsangbuk province Cheongdo, KBES—Kyeongsangbuk province Uiseong, KBYC—Kyeongsangbuk province Yeongcheon, KNCN—Kyeongsangnam province Changnyeong, KNMY—Kyeongsangnam province Miryang, KNGC—Kyeongsangnam province Geochang.

When the multivariate statistical analysis was performed only on domestic samples in Korea to investigate the possibility of our method to the discrimination of samples cultivated in the regions close to each other, it could not distinguish soybeans according to the region in the results of PCA (data not shown) and PLS-DA. Figure 3a shows that PLS 1, 2, and 3 together explained 43.7% of the total variance (19.09%, 15.36%, and 8.92%, respectively), while Figure 3b shows that two PLS components (PLS components 1 and 2) explained 33.86%. The parameters of the cross-validation modeling were component 3, with R^2^X = 0.437, R^2^Y = 0.169, and Q^2^(cum) = 0.0535. After 100 times permutations, R^2^ = 0.0951 and Q^2^ = −0.0676 were obtained.

Soybean samples from the Gyeonggi and Kyeongsangbuk provinces were clustered according to their regions, whereas other samples were not clearly clustered in the PLS-DA score plot. As shown in Table 1, the climatic conditions varied with the cultivation area. The mean temperatures in 2016 showed similar tendencies in all of the cultivation regions studied, but with slight differences in the total precipitation and sun exposure times. Various plant volatiles can be affected by changing biotic and abiotic factors [54]. Vallat et al. explained that the concentrations of nonanal and benzaldehyde were both positively related to precipitation, and positively and negatively related to temperature, respectively [54]. This variety of climate factors could together affect the volatile metabolites formed in soybeans cultivated in different regions. However, the relationships between climate and the amounts of nonanal and benzaldehyde formed were not clear in this study. Other domestic samples except those from the Gyeonggi and Kyeongsangbuk provinces were not clearly grouped in the PLS-DA score plot.

## 3. Materials and Methods

### 3.1. Materials

Thirty-six different soybean samples (17 from Korea, 13 from China, and 6 from North America) cultivated in 2016 (Figures S1–S3, Table 4) were used. Soybeans from Korea were provided by the National Agricultural Products Quality Management Service, whereas those from China were obtained from Chinese markets (Figures S4 and S5, Table S4). Soybeans from North America were gifts from a soybean processing company in Korea (Figure S6, Table S4). All samples were stored at −70 °C in a deep freezer before they were analyzed. Solid-phase microextraction (SPME) fibers and holders were purchased from Supelco (Bellefonte, PA, USA), whereas vials and screw caps (Ultraclean 18 mm) were purchased from Agilent Technologies (Santa Clara, CA, USA). l-Borneol was purchased from Sigma-Aldrich (St. Louis, MO, USA). Authentic standard compounds for positive identification of volatile compounds were purchased as follows: 3-methylphenol and hexan-1-ol were purchased from Supelco (Bellefonte, PA, USA), 1,3-benzothiazole, acetaldehyde, α-terpinene were obtained from Fluka (St. Gallen, Switzerland), and acetonitrile was bought from J.T. Baker (Phillipsburg, NJ, USA), while all of the other authentic standards were purchased from Sigma-Aldrich (St. Louis, MO, USA).

### 3.2. Extraction of Volatile Metabolites Using SPME

l-Borneol was prepared at 200 mg/L with *tert*-butanol. Then, distilled water was added at a final concentration of 1 mg/L before soybean (5 g) was placed in a 20 mL screw vial with a screw cap. SPME was used to obtain volatile metabolites of soybeans. The sample was maintained at 40 °C for 30 min to reach the equilibrium state. SPME fiber coated with carboxen/polydimethylsiloxane/ divinylbenzene (CAR/PDMS/DVB) was used to adsorb volatile compounds at 40 °C for 20 min, and desorption was executed at 200 °C in a GC injector for 5 min while cryo-trapping at −80 °C. For every other ten runs in GC-MS analysis, we included quality control (QC) soybean samples to confirm the relative peak areas and retention times of several main volatile compounds.

### 3.3. GC-MS Analysis

The GC-MS analysis was performed using a 7890A series gas chromatograph (Agilent Technologies, Santa Clara, CA, USA) and a 5975C mass detector (Agilent Technologies, Santa Clara, CA, USA) equipped with a DB-Wax column (30 m length × 0.25 mm i.d. × 0.25 μm film thickness, J&W Scientific, Folsom, CA, USA). GC oven temperature was programmed as follows; initial temperature was maintained 40 °C for 10 min, raised to 42 °C at a rate of 2 °C/min and held for 3 min, and increased to 100 °C at a rate of 4 °C/min and kept for 5 min, and raised 180 °C at a rate of 4 °C/min, and the ramped to 200 °C at a rate of 10 °C/min. The flow rate of helium, carrier gas, was constant at 0.8 mL/min, whereas mass spectra were obtained with a mass scan rage of 35–350 atomic mass unites (a.m.u.) at a rate of 4.5 scans/sec, and the electron impact (EI) mode was 70 eV. All sample preparations and analyses were independently performed in triplicate. In the preliminary study, we confirmed the repeatability and precision of our method on the results of the main volatile compounds in soybean in more than six replicates.

### 3.4. Identification and Quantification of Volatile Metabolites

The identification of each volatile compound was positively confirmed by comparison of retention time and mass spectral data with those of authentic standard compounds. When standard compounds were not available, each volatile compound was identified on the basis of its mass spectral data using the NIST.08 and Wiley.9 mass spectral libraries and the retention index (RI) values in the previous literature. The RI value of volatile compounds was calculated with *n*-alkane from C_6_ to C_30_ as an external standard. The quantification of the volatile components was calculated to obtain relative peak areas by comparing their peak areas with that of the internal standard compound on the total ion chromatogram of GC-MS. Five microliters of l-borneol (1 mg/L in *tert*-butanol/distilled water solvents mixture (1:200, *v*/*v*)) was used as an internal standard.

### 3.5. Statistical Analysis

All the datasets obtained were processed by multivariate statistical analysis, such as principal components analysis (PCA) and partial least square-discriminant analysis (PLS-DA) using SIMCA-P (version 11.0, Umetrics, Umea, Sweden), to determine the discrimination of soybeans according to different geographic origins. Heatmap visualization and hierarchical clustering analysis were performed based on Pearson’s correlation and average linkage method using Multi Experimental Viewer (MeV) software (version 4.9, The Institute for Genomic Research (TIGR)) [55].

## 4. Conclusions

This study applied GC-MS analysis combined with the multivariate statistical analysis to distinguish the geographical origins of soybeans. The profiles of volatile compounds in the soybean samples varied with their cultivation regions. In the PLS-DA results, all soybean samples were clearly discriminated by their geographical origins. However, those cultivated in Korea (except for the samples from the Gyeonggi and Kyeongsangbuk provinces) could not be clearly separated according to the region on the PLS-DA score plot. We also determined the major volatile metabolites that contributed to the discrimination of geographical origins on the basis of PLS-DA. This study has the advantage of being able to distinguish the geographical origin of soybeans without any sample pretreatment on the basis of volatile metabolite profiles, which are highly related to their quality. However, we did not have enough sample information on post-harvest practices, such as drying and storage conditions, which could affect volatiles’ profiles in some way. Nevertheless, our result could be applied to the discrimination of soybeans distributed and commercially available in Korea, the main objective of this study.

In summary, the findings of this study suggested that combining GC-MS-based analysis of volatile compounds with multivariate data analysis is a useful tool for discriminating the geographical origins of soybeans, but with some limitations for domestically cultivated ones.

## Figures and Tables

**Table 1:** Climatic conditions and geographic coordinates of Korea, China, and North America.

Province	Latitude	Longitude	Annual Mean Temperature (°C)	Annual Mean Precipitation (mm)
**Korea**				
Gyeongi	11° 7′ 15.744″ N	105° 32′ 0.5748″ E	11.7	1240
Gangwon	37° 52′ 52.7268″ N	37° 52′ 52.7268″ N	10.9	1307
Chungcheongbuk	36° 56′ 10.068″ N	127° 41′ 44.736″ E	10.8	1239
Chungcheongnam	36° 48′ 33.5196″ N	127° 9′ 36.1512″ E	11.8	1229
Jeollabuk	35° 47′ 52.8432″ N	126° 53′ 31.9632″ E	12.8	1251
Jeollnam	35° 1′ 37.308″ N	126° 43′ 15.024″ E	13.9	1264
Kyeongsangbuk	35° 59′ 18.312″ N	128° 56′ 31.2″ E	12.6	1026
Kyeongsangnam	35° 31′ 48.792″ N	128° 30′ 28.116″ E	13.3	1248
**North America**				
Illinois	40° 37′ 59.25″ N	89° 23′ 54.7044″ W	11.42	947.93
Indiana	40° 16′ 1.8948″ N	86° 8′ 5.6508″ W	11.1	1011
Minnesota	46° 43′ 46.3908″ N	94° 41′ 9.2328″ W	7.3	807
Michigan	44° 18′ 53.4312″ N	85° 36′ 8.5104″ W	8.6	890
Quebec	52° 56′ 23.694″ N	73° 32′ 56.8788″ W	4.8	1001
Ontario	51° 15′ 13.5972″ N	85° 19′ 23.5632″ W	7.1	775.9
**China**				
Heilongjian	45° 37′ 17.9832″ N	126° 14′ 35.3466″ E	3.4	562
Jilin	42° 31′ 40.44″ N	125° 40′ 40.7994″ E	4.9	784
Liaoning	40° 1′ 44.1114″ N	124° 17′ 4.4484″ E	9.0	1040
Hebei	38° 16′ 53.5578″ N	114° 41′ 29.7276″ E	13.2	517
Shandong	41° 1′ 59.0874″ N	113° 6′ 25.6314″ E	14.1	676
Hubei	30° 13′ 35.3634″ N	115° 3′ 49.4634″ E	17.0	1396
Anhui	33° 57′ 22.248″ N	116° 47′ 20.5434″ E	15.2	728
Zhejiang	30° 42′ 1.8″ N	121° 0′ 37.3314″ E	16.2	1118
Fujian	25° 6′ 50.796″ N	99° 9′ 44.28″ E	20.7	1677
Jiangxi	31° 21′ 54.6474″ N	118° 23′ 22.8114″ E	17.2	1475
Guangdong	24° 48′ 4.068″ N	113° 35′ 33.7554″ E	21.0	1499

**Table 2:** The major volatile metabolites identified in soybeans from Korea, China, and North America according to variables importance plot (VIP > 1.20) list for partial least square (PLS) component 1.

Retention Index (RI) Cal ^1^	RI Ref ^2^	Volatile Compounds	VIP Values	Identification (ID) ^3^
Negative direction					
1289	1288	Heptan-4-ol	2.29	B
1151		Butan-1-ol	2.19	A
1217		Butyl butanoate	2.00	A
1285	1287	Octanal	1.97	B
1175		Butyl prop-2-enoate	1.89	A
1642	1631	5-Methyl-2-propan-2-ylcyclohexan-1-ol	1.88	B
1067		Butyl acetate	1.88	A
1141		Butyl propanoate	1.84	A
1391		Nonanal	1.83	A
1027		Toluene	1.80	A
1181		Heptanal	1.75	A
1122		Heptan-4-one	1.74	A
1688	1694	5-Ethyloxolan-2-one	1.67	B
1273		1,2,3-Trimethylbenzene	1.63	A
1178		Heptan-2-one	1.55	A
995		Acetonitrile	1.54	A
1210		*(E)*-Hex-2-enal	1.54	A
1234		Ethyl hexanoate	1.49	A
1317		*(E*)-Hept-2-enal	1.45	A
1190		Limonene	1.40	A
959	968	1-Butoxybutane	1.37	B
1230		2-Pentylfuran	1.30	A
605		Acetaldehyde	1.27	A
1162		Myrcene	1.27	A
1279		3-Hydroxybutan-2-one	1.22	A
Positive direction					
1493		2-Ethylhexan-1-ol	1.75	A

^1^ Retention indices were determined using *n*-alkanes C_6_ to C_30_ as an external standard; ^2^ Retention indices were obtained from national institute of standards and technology (NIST) database (http://webbook.nist.gov/chemistry); ^3^ Identification of the compounds was based as follows; A, mass spectrum and retention index agree with the authentic compounds under similar conditions (positive identification); B, mass spectrum and retention index were consistent with those from NIST database.

**Table 3:** The major volatile metabolites identified in soybeans from Korea, China, and North America according to variables importance plot (VIP > 1.20) list for partial least square PLS component 2.

RI Cal ^1^	RI Ref ^2^	Volatile Compounds	VIP Values	ID ^3^
Negative direction				
1493		2-Ethylhexan-1-ol	2.73	A
1194		2,5-Dimethylhexan-2-ol	2.29	C
1250		Styrene	2.16	A
850		2-Methylfuran	2.02	A
<600		2-Methylprop-1-ene	1.93	C
792		Propan-2-one	1.88	A
861		2-Methylprop-2-enal	1.85	A
600		Hexane	1.80	A
810		Methyl acetate	1.48	A
1304	1312	2-Methylpentan-1-ol	1.40	B
1285	1287	Octanal	1.37	B
1175		Butyl prop-2-enonate	1.29	A
1129		1-Methoxypropan-2-ol	1.23	A
1181		Heptanal	1.22	A
1027		Toluene	1.21	A
Positive direction				
1449		Oct-1-en-3-ol	2.32	A
900	900	Nonane	2.11	B
1598		4-Methyloxolan-2-one	1.67	C
1289	1288	Heptan-4-ol	1.54	B
1151		Butan-1-ol	1.48	A
1252		Octan-3-one	1.43	A
1220		Butyl butanoate	1.35	A
1850		3-Hydroxy-2,4,4-trimethylpentyl 2-methylpropanoate	1.33	C
1642	1631	5-Methyl-2-propan-2-ylcyclohexan-1-ol	1.26	B
1067		Butyl acetate	1.26	A
1140		Butyl propanoate	1.24	A
1391		Nonanal	1.23	A

^1^ Retention indices were determined using *n*-alkanes C_6_ to C_30_ as an external standard; ^2^ Retention indices were obtained from NIST database (http://webbook.nist.gov/chemistry); ^3^ Identification of the compounds was based as follows; A, mass spectrum and retention index agree with the authentic compounds under similar conditions (positive identification); B, mass spectrum and retention index were consistent with those from NIST database; C, mass spectrum was consistent with that of W9N08 (Wiley and NIST) and manual interpretation (tentative identification).

**Table 4:** The origins of the 36 soybean samples from Korea, China, and North America.

Nation	Province	Location	Labeling ^1^
**Korea**	Gyeonggi	Anseong	GGIC
		Icheon	GGAS
	Gangwon	Chuncheon	GWCC
		Yeongwol	GWYW
	Chungcheongbuk	Eumseong	CBES
	Chungcheongnam	Cheonan	CNCA
		Gongju	CNGJ
	Jeollabuk	Gimje	JBGJ
		Imsil	JBIS
	Jeollanam	Naju	JNNJ
		Yeonggwang	JNYG
	Kyeongsangbuk	Cheongdo	KBCD
		Uiseong	KBES
		Yeongcheon	KBYC
	Kyeongsangnam	Changnyeong	KNCN
		Miryang	KNMY
		Geochang	KNGC
**China**	Neimenggu	Ulanhot	INUL
	Heilongjiang	Harbin	HEHA
	Jilin	Meihekou	JIME
	Liaoning	Dandong	LIDA
	Hebei	Shijiazhuang	HESH
	Shandong	Jining	SHJI
	Anhui	Huaibei	ANHU
	Hubei	Huangshi	HUHU
	Zhejiang	Pinghu	ZHPI
	Jiangxi	Jiujiang	JIJI
	Fujian	Longyan	FULO
	Guangdong	Shaoguan	GUSH
	Guangxi	Hechi	GUBA
**The United States (North America)**	Illinois		IL
	Indiana		IN
	Minnesota		MN
	Michigan		MI
**Canada (North America)**	Quebec		QB
	Ontario		ON

^1^ GGIC—Gyeonggi province Anseong, GGAS—Gyeonggi province Icheon, GWCC—Gangwon province Chuncheon, GWYW—Gangwon province Yeongwol, CBES—Chungcheongbuk province Eumseong, CNCA— Chungcheongnam province Cheonan, CNGJ—Chungcheongnam province Gongju, JBGJ—Jeollabuk province Gimje, JBIS—Jeollabuk province Imsil, JNNJ—Jeollanam province Naju, JNYG—Jeollanam province Yeonggwang, KBCD—Kyeongsangbuk province Cheongdo, KBES—Kyeongsangbuk province Uiseong, KBYC—Kyeongsangbuk province Yeongcheon, KNCN—Kyeongsangnam province Changnyeong, KNMY—Kyeongsangnam province Miryang, KNGC—Kyeongsangnam province Geochang, INUL—Neimenggu province Ulanhot, HEHA—Heilongjiang province Harbin, JIME—Jilin province Meihekou, LIDA—Liaoning province Dandong, HESH—Hebei province Shijiazhuang, SHJI—Shandong province Jining, ANHU—Anhui province Huaibei, HUHU—Hubei province Huangshi, ZHPI—Zhejiang province Pinghu, JIJI—Jiangxi province Jiujiang, FULO—Fujian province Longyan, GUSH—Guangdong province Shaoguan, GUBA—Guangxi province Hechi, IL—Illinois province, IN—Indiana province, MN—Minnesota province, MI—Michigan province, QB—Quebec province, ON—Ontario province.

## Supplementary Materials

The following are available online at https://www.mdpi.com/1420-3049/25/3/763/s1, Figures S1–S6, Tables S1–S4.
