# Supplementary material for: Discrimination of Cultivated Regions of Soybeans (Glycine max) Based on Multivariate Data Analysis of Volatile Metabolite Profiles
Source: Molecules. 2020 Feb 10;25(3):763. doi: 10.3390/molecules25030763 (PMC7036852; doi:10.3390/molecules25030763)
Supplement: Supplementary file 1 [file molecules-25-00763-s001.pdf]

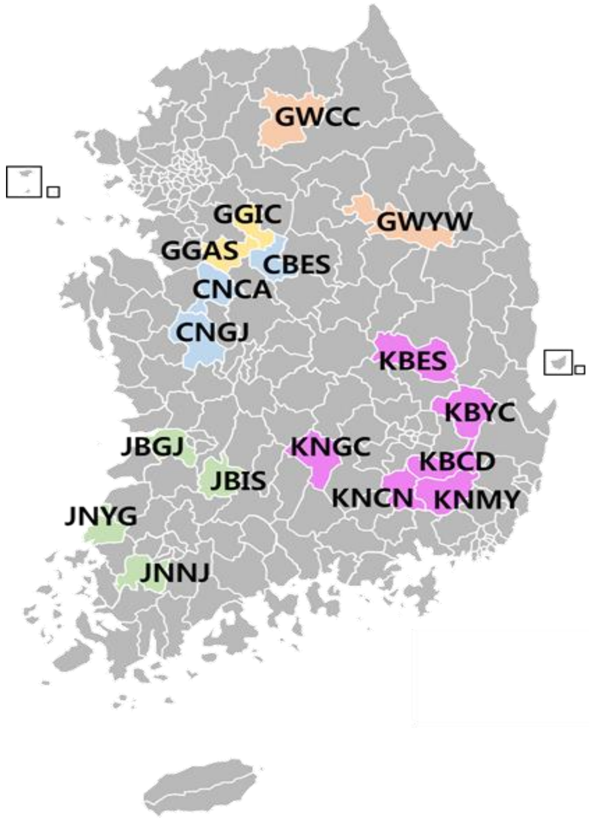

Figure S1: Map of Korea

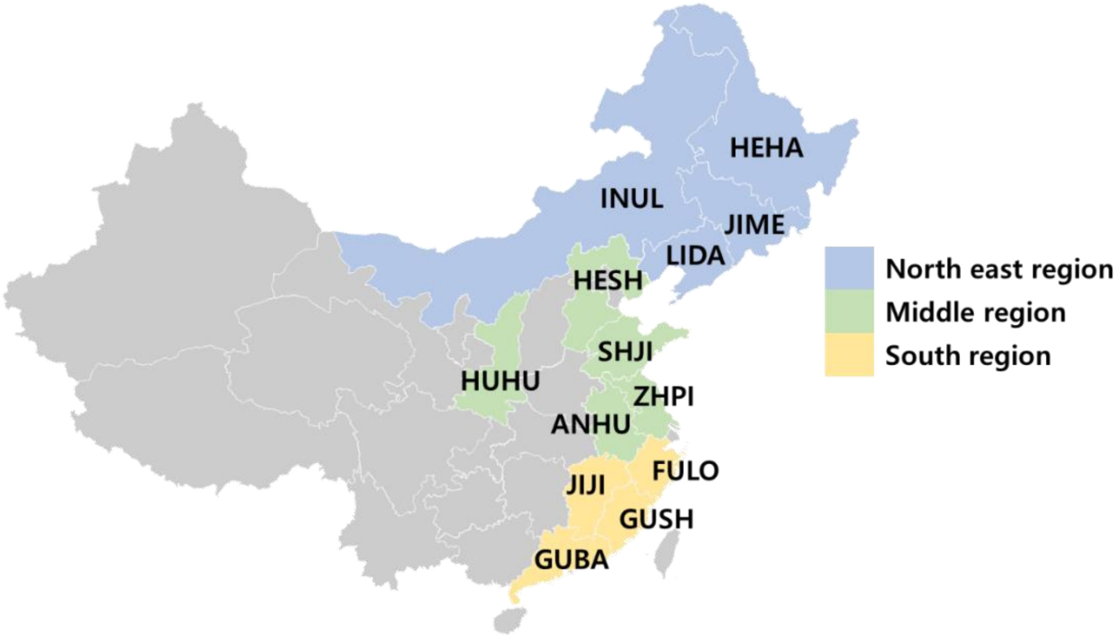

Figure S2: Map of China

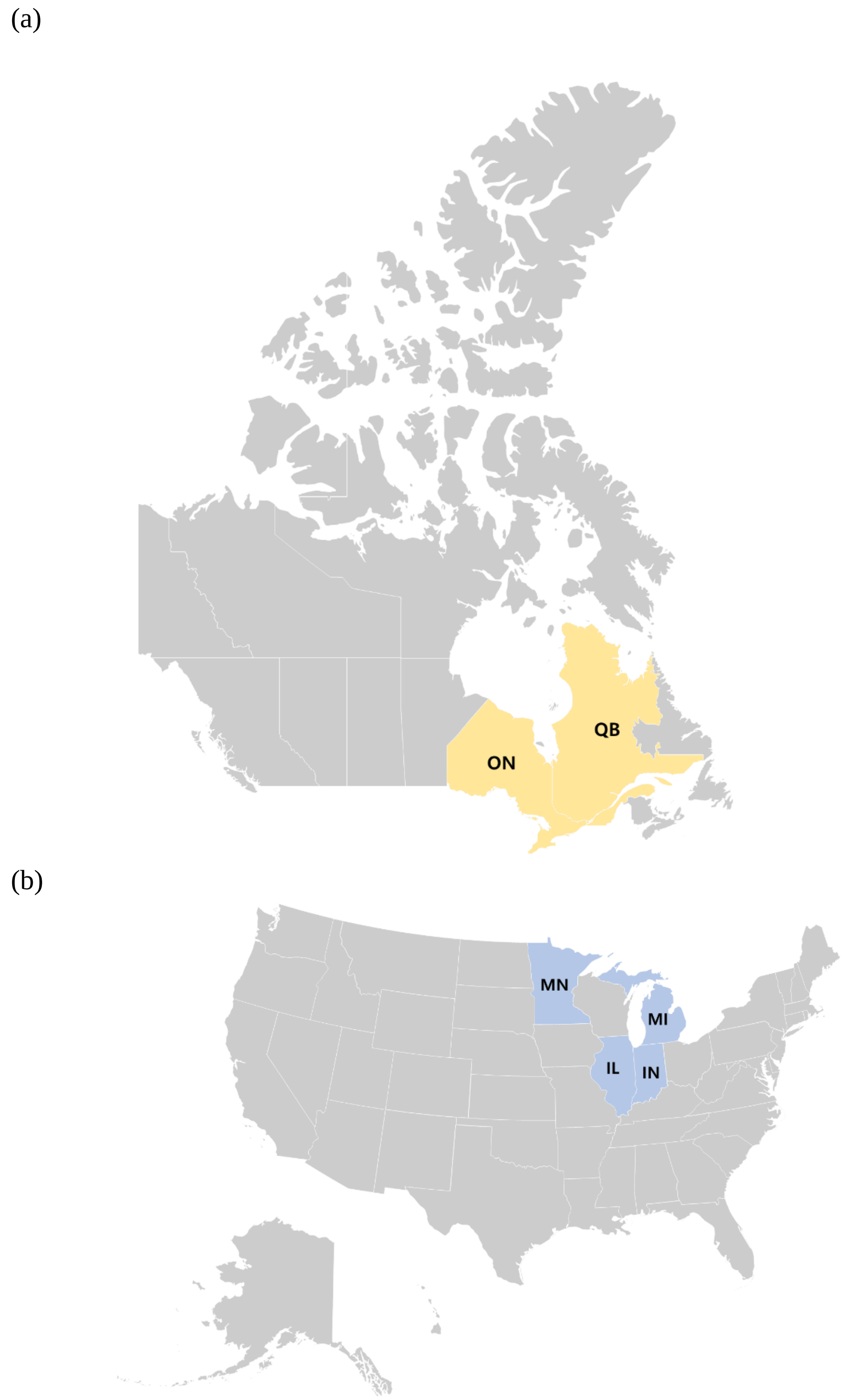

Figure S3: Map of North America: (a) Canada, (b) the United States.

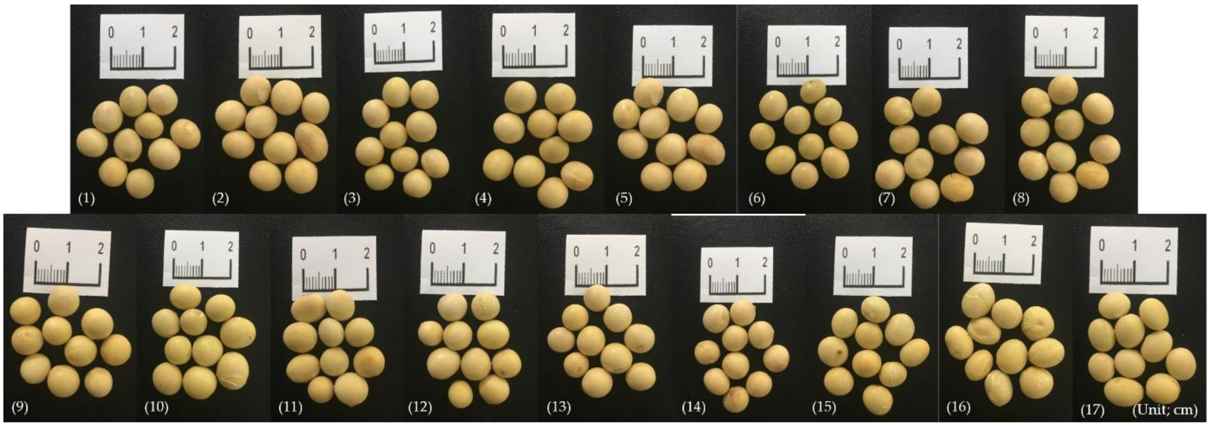

Fig S4: Morphological characteristics of the 17 soybean samples cultivation from Korea.; (1) Gyeonggi province Anseong; (2) Gyeonggi province Icheon; (3) Gangwon province Chuncheon; (4) Gangwon province Yeongwol; (5) Chungcheongbuk province Eumseong; (6) Chungcheongnam province Cheonan; (7) Chungcheongnam province Gonju; (8) Jeollabuk province Gimje; (9) Jeollabuk province Imsil; (10) Jeollanam province Naju; (11) Jeollanam province Yeonggwang; (12) Kyeongsangbuk province Cheongdo; (13) Kyeongsangbuk province Uiseong; (14) Kyeongsangbuk province Yeongcheon; (15) Kyeongsangnam province Changnyeong; (16) Kyeongsangnam province Miryang; (17) Kyeongsangnam province Geochang.

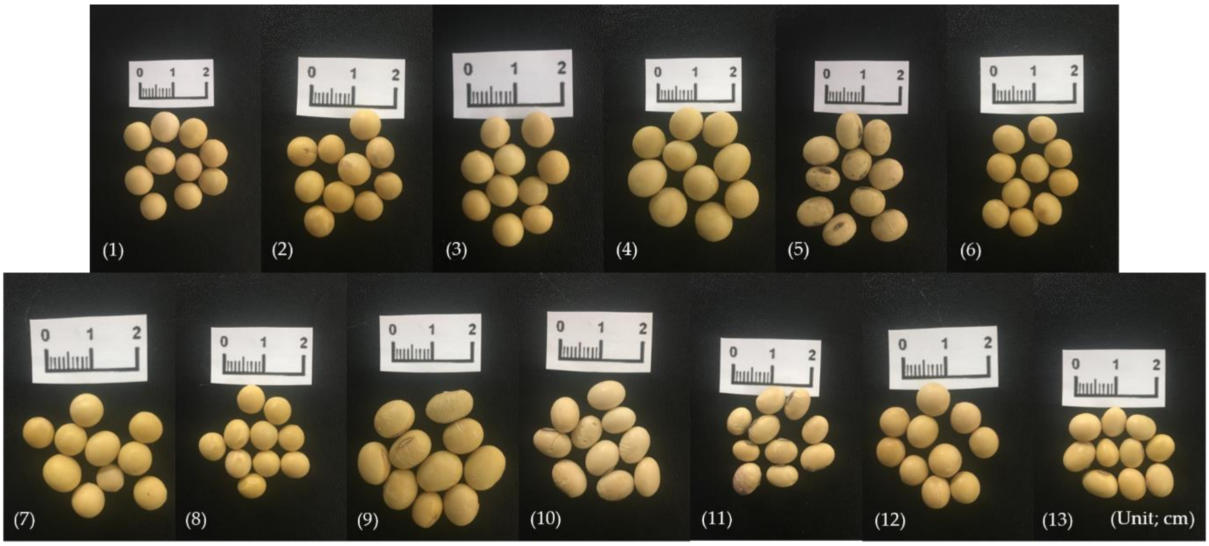

Fig S5: Morphological characteristics of the 13 soybean samples cultivation from China.; (1) Neimenggu province Ulanhot; (2) Heilongjiang province Harbin; (3) Jilin province Meihekou; (4) Liaoning province Dandong; (5) Hebei province Shijiazhuang; (6) Shandong province Jining; (7) Anhui province Huaibei; (8) Hubei province Huangshi; (9) Zhejiang province Pinghu; (10) Jiangxi province Jiujiang; (11) Fujian province Longyan; (12) Guangdong province Shaoguan; (13) Guangxi province Hechi.

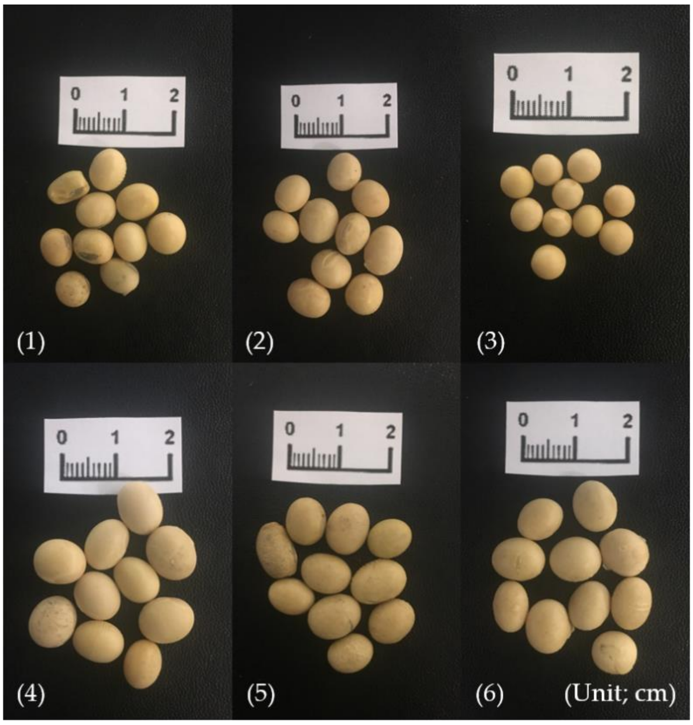

Fig S6: Morphological characteristics of the 6 soybean samples cultivation from North America.; (1) Illinois province; (2) Indiana province; (3) Minnesota province; (4) Michigan province; (5) Quebec province; (6) Ontario province.

Table S1: Volatile metabolites of soybeans cultivated in Korea

No.[1]	RI cal[2]	RI ref[3]	Volatile Compounds	Relative Peak Area[4]																	ID[6]
				CBES[7]	CNCA	CNGJ	GGAS	GGIC	GWCC	GWYW	JBGJ	JBIS	JNNJ	JNYG	KBCD	KBES	KBYC	KNCN	KNGC	KNMY	
			**Acids**																		
1	1449		Acetic acid	1.214 ±0.100	1.03 ±0.057	0.284 ±0.057	1.1 ±0.211	1.491 ±0.080	1.167 ±0.160	0.898 ±0.212	0.711 ±0.057	0.384 ±0.136	1.241 ±0.708	0.404 ±0.039	0.202 ±0.039	N.D.[5]	0.376 ±0.126	1.181 ±0.065	0.377 ±0.151	0.098 ±0.013	A
			**Alcohols**																		
2	924		Propan-2-ol	N.D.	N.D.	0.045 ±0.007	N.D.	N.D.	N.D.	0.165 ±0.233	N.D.	N.D.	0.045 ±0.043	N.D.	N.D.	N.D.	N.D.	N.D.	0.067 ±0.022	N.D.	A
3	928		Ethanol	1.293 ±0.091	2.099 ±0.267	1.852 ±0.335	1.285 ±0.18	0.495 ±0.031	2.497 ±0.323	5.416 ±0.462	4.1 ±0.793	2.928 ±1.975	5.302 ±2.205	2.052 ±0.298	N.D.	0.351 ±0.049	1.673 ±0.718	N.D.	3.123 ±0.756	1.473 ±0.22	A
4	1024		Butan-2-ol	1.089 ±0.189	N.D.	1.439 ±0.146	N.D.	1.291 ±0.029	1.898 ±0.298	1.466 ±0.157	N.D.	N.D.	N.D.	1.248 ±0.079	N.D.	N.D.	0.107 ±0.185	N.D.	N.D.	N.D.	A
5	1101		2-Methylpropan-1-ol	N.D.	N.D.	N.D.	N.D.	N.D.	N.D.	0.224 ±0.006	N.D.	N.D.	N.D.	N.D.	N.D.	0.063 ±0.011	N.D.	N.D.	N.D.	N.D.	A
6	1129		1-Methoxypropan-2-ol	1.464 ±0.093	1.579 ±0.03	0.769 ±0.103	0.75 ±0.085	1.655 ±0.09	1.233 ±0.097	0.293 ±0.046	0.551 ±0.025	1.292 ±0.45	1.449 ±0.33	1.083 ±0.07	1.309 ±0.428	0.222 ±0.037	0.975 ±0.257	0.443 ±0.119	0.709 ±0.091	3.063 ±0.34	A
7	1151		Butan-1-ol	0.81 ±0.096	0.279 ±0.038	0.368 ±0.038	0.193 ±0.04	0.745 ±0.074	0.649 ±0.313	1.009 ±0.109	0.188 ±0.009	N.D.	0.329 ±0.141	0.369 ±0.009	3.35 ±1.359	N.D.	0.108 ±0.188	0.499 ±0.136	0.165 ±0.052	0.493 ±0.118	A
8	1194		2,5-Dimethylhexan-2-ol	0.274 ±0.006	0.227 ±0.021	0.224 ±0.025	N.D.	N.D.	0.258 ±0.027	0.275 ±0.034	0.217 ±0.011	0.233 ±0.009	0.27 ±0.087	0.23 ±0.021	0.226 ±0.144	0.356 ±0.035	0.174 ±0.302	0.293 ±0.013	0.313 ±0.031	0.344 ±0.059	C
9	1211		3-Methylbutan-1-ol	N.D.	N.D.	N.D.	N.D.	N.D.	N.D.	1.241 ±0.019	0.537 ±0.05	N.D.	4.1 ±5.626	N.D.	N.D.	0.645 ±0.091	N.D.	1.05 ±0.059	N.D.	N.D.	A
10	1255		Pentan-1-ol	0.367 ±0.019	0.723 ±0.25	0.502 ±0.038	0.507 ±0.18	0.136 ±0.011	0.333 ±0.018	0.301 ±0.055	0.424 ±0.011	N.D.	1.979 ±1.892	0.332 ±0.03	0.383 ±0.137	0.274 ±0.021	0.476 ±0.287	0.493 ±0.162	0.409 ±0.086	0.728 ±0.184	A
11	1304		2-Methylpentan-1-ol	N.D.	0.152 ±0.027	N.D.	N.D.	N.D.	0.087 ±0.007	0.129 ±0.002	0.069 ±0.002	N.D.	0.194 ±0.105	N.D.	N.D.	N.D.	0.21 ±0.098	N.D.	N.D.	N.D.	B
12	1344		Hexan-1-ol	0.568 ±0.052	1.777 ±0.467	1.566 ±0.226	0.813 ±0.491	0.071 ±0.007	0.209 ±0.021	1.001 ±0.286	1.54 ±0.100	0.604 ±0.228	6.45 ±7.518	0.585 ±0.07	0.465 ±0.092	1.252 ±0.08	0.611 ±0.65	1.938 ±0.632	0.641 ±0.178	2.145 ±0.046	A
13	1402		2-Butoxyethanol	N.D.	N.D.	0.059 ±0.012	N.D.	0.054 ±0.007	N.D.	N.D.	N.D.	0.079 ±0.0100	N.D.	N.D.	N.D.	N.D.	N.D.	0.089 ±0.043	N.D.	0.1 ±0.035	A
14	1449		Oct-1-en-3-ol	N.D.	N.D.	N.D.	N.D.	N.D.	N.D.	N.D.	N.D.	N.D.	N.D.	N.D.	N.D.	0.674 ±0.035	N.D.	N.D.	N.D.	N.D.	A
15	1493		2-Ethylhexan-1-ol	8.806 ±0.692	10.443 ±0.929	13.104 ±0.546	9.74 ±1.446	5.697 ±0.23	6.864 ±0.262	10.167 ±0.402	8.925 ±0.309	8.434 ±2.989	11.863 ±1.932	5.995 ±0.502	7.06 ±1.883	2.517 ±0.445	4.624 ±1.299	3.85 ±3.381	14.73 ±0.700	8.315 ±0.577	A
16	1565		Octan-1-ol	N.D.	N.D.	N.D.	0.114 ±0.027	0.043 ±0.004	N.D.	N.D.	N.D.	N.D.	N.D.	N.D.	0.098 ±0.041	N.D.	0.095 ±0.015	0.102 ±0.017	N.D.	0.254 ±0.021	A
17	1582	1580	Butane-2,3-diol	0.198 ±0.021	0.092 ±0.024	N.D.	N.D.	N.D.	N.D.	N.D.	N.D.	N.D.	0.366 ±0.560	N.D.	N.D.	N.D.	N.D.	N.D.	N.D.	N.D.	B
18	1643	1631	5-Methyl-2-propan-2-ylcyclohexan-1-ol	N.D.	0.104 ±0.011	N.D.	0.068 ±0.06	0.061 ±0.002	N.D.	N.D.	0.064 ±0.020	N.D.	N.D.	N.D.	N.D.	N.D.	N.D.	0.32 ±0.044	N.D.	N.D.	B
19	1669		Nonan-1-ol	N.D.	N.D.	N.D.	N.D.	N.D.	N.D.	N.D.	0.18 ±0.017	N.D.	N.D.	N.D.	N.D.	N.D.	0.055 ±0.048	N.D.	N.D.	0.141 ±0.032	A

20	1759		2-Phenylpropan-2-ol	N.D.	N.D.	N.D.	N.D.	N.D.	N.D.	N.D.	N.D.	N.D.	N.D.	N.D.	N.D.	0.166 ±0.029	0.238 ±0.05	N.D.	N.D.	0.422 ±0.009	A
21	1832		1-Phenylethanol	N.D.	N.D.	N.D.	N.D.	N.D.	N.D.	N.D.	N.D.	N.D.	N.D.	N.D.	N.D.	N.D.	N.D.	N.D.	N.D.	0.218 ±0.012	A
22	1877		Phenylmethanol	0.161 ±0.021	0.122 ±0.008	N.D.	0.168 ±0.026	N.D.	0.1 ±0.016	N.D.	N.D.	N.D.	0.3 ±0.073	0.148 ±0.007	N.D.	N.D.	N.D.	N.D.	N.D.	0.179 ±0.311	A
23	1898		2-Phenylethanol	N.D.	N.D.	N.D.	N.D.	N.D.	N.D.	N.D.	N.D.	N.D.	N.D.	N.D.	N.D.	N.D.	N.D.	0.019 ±0.002	N.D.	0.004 ±0.006	A
24	2087	2087	2-(Phenoxy)ethanol	N.D.	N.D.	N.D.	N.D.	N.D.	N.D.	N.D.	N.D.	N.D.	N.D.	N.D.	N.D.	N.D.	N.D.	0.014 ±0.005	N.D.	N.D.	B
			**Aldehydes**																		
25	605		Acetaldehyde	N.D.	0.11 ±0.018	0.029 ±0.002	N.D.	0.049 ±0.016	0.064 ±0.017	0.081 ±0.025	N.D.	0.106 ±0.01	N.D.	0.071 ±0.019	N.D.	N.D.	N.D.	N.D.	0.073 ±0.01	N.D.	A
26	861		2-Methylprop-2-enal	0.015 ±0.001	0.017 ±0.002	0.018 ±0.001	0.018 ±0.004	0.011 ±0.001	0.013 ±0.003	0.02 ±0.007	0.017 ±0.001	0.018 ±0.005	0.035 ±0.010	0.012 ±0.003	N.D.	N.D.	N.D.	N.D.	0.025 ±0.005	N.D.	A
27	968		Pentanal	N.D.	N.D.	N.D.	N.D.	N.D.	N.D.	N.D.	N.D.	0.13 ±0.110	N.D.	N.D.	N.D.	N.D.	N.D.	N.D.	N.D.	N.D.	A
28	1074		Hexanal	0.604 ±0.097	4.607 ±4.338	1.973 ±0.857	3.815 ±4.195	0.324 ±0.034	0.834 ±0.042	0.555 ±0.167	1.052 ±0.076	4.518 ±1.02	2.108 ±2.591	0.445 ±0.388	3.055 ±0.857	1.253 ±0.372	3.224 ±3.711	N.D.	N.D.	N.D.	A
29	1181		Heptanal	N.D.	0.264 ±0.077	0.198 ±0.021	0.3 ±0.080	0.102 ±0.010	0.149 ±0.026	N.D.	0.171 ±0.011	N.D.	N.D.	0.105 ±0.008	0.005 ±0.008	0.133 ±0.028	0.42 ±0.290	N.D.	N.D.	N.D.	A
30	1210		(*E*)-Hex-2-enal	N.D.	N.D.	N.D.	0.255 ±0.033	N.D.	N.D.	N.D.	N.D.	N.D.	N.D.	N.D.	0.103 ±0.090	N.D.	N.D.	N.D.	N.D.	N.D.	A
31	1285	1287	Octanal	0.057 ±0.019	0.12 ±0.051	0.093 ±0.009	0.349 ±0.083	0.12 4±0.02	0.071 ±0.005	0.068 ±0.029	0.187 ±0.025	0.107 ±0.02	0.123 ±0.013	0.094 ±0.003	0.174 ±0.034	0.156 ±0.027	0.407 ±0.363	0.16 ±0.009	0.234 ±0.017	0.247 ±0.099	B
32	1391		Nonanal	0.421 ±0.159	0.663 ±0.189	0.601 ±0.012	1.668 ±0.256	0.674 ±0.101	0.628 ±0.059	0.333 ±0.196	0.959 ±0.063	0.796 ±0.165	1.093 ±0.172	0.68 ±0.107	1.136 ±0.267	0.767 ±0.192	2.783 ±1.754	0.836 ±0.079	1.161 ±0.1	1.782 ±0.762	A
33	1515		Benzaldehyde	0.191 ±0.008	0.198 ±0.027	0.197 ±0.044	0.217 ±0.021	0.119 ±0.012	0.152 ±0.004	N.D.	N.D.	0.178 ±0.023	0.241 ±0.074	0.204 ±0.016	0.382 ±0.088	0.094 ±0.007	0.595 ±0.237	0.114 ±0.005	0.122 ±0.021	0.693 ±0.062	A
			**Benzenes**																		
34	1119		Ethylbenzene	N.D.	0.094 ±0.063	0.148 ±0.132	N.D.	N.D.	0.055 ±0.005	0.48 ±0.053	0.061 ±0.01	0.031 ±0.053	0.475 ±0.217	0.064 ±0.009	0.164 ±0.064	0.205 ±0.294	0.925 ±0.581	0.152 ±0.042	N.D.	1.326 ±0.302	A
35	1126		1,4-Xylene	N.D.	N.D.	0.022 ±0.038	N.D.	N.D.	N.D.	0.345 ±0.038	N.D.	N.D.	N.D.	N.D.	N.D.	0.013 ±0.023	N.D.	N.D.	N.D.	N.D.	A
36	1133		1,3-Xylene	N.D.	N.D.	0.078 ±0.135	N.D.	N.D.	N.D.	1.24 5±0.112	N.D.	N.D.	N.D.	0.009 ±0.008	0.146 ±0.094	0.182 ±0.083	0.129 ±0.069	0.138 ±0.068	0.582 ±0.215	0.128± 0.026	A
37	1175		1,2-Xylene	N.D.	N.D.	0.042 ±0.072	N.D.	N.D.	N.D.	1.613 ±0.159	N.D.	N.D.	N.D.	0.043 ±0.002	N.D.	0.109 ±0.056	0.048 ±0.023	N.D.	1.027 ±0.28	0.086 ±0.035	A
38	1250		Styrene	0.355 ±0.033	0.725 ±0.209	0.532 ±0.148	0.66 ±0.111	0.165 ±0.034	0.392 ±0.04	0.963 ±0.116	0.317 ±0.027	0.424 ±0.256	2.647 ±0.89	0.461 ±0.041	0.55 ±0.232	0.801 ±0.141	1.618 ±0.956	0.382 ±0.328	0.708 ±0.114	1.588 ±0.371	A
39	1264		1-Methyl-3-propan-2-ylbenzene	0.036 ±0.006	N.D.	N.D.	N.D.	N.D.	N.D.	N.D.	N.D.	N.D.	0.25 ±0.248	N.D.	N.D.	N.D.	N.D.	N.D.	N.D.	N.D.	A

			**Esters**																	
40	810		Methyl acetate	0.257 ±0.023	0.187 ±0.005	0.151 ±0.066	0.208 ±0.017	N.D.	0.316 ±0.067	0.481 ±0.038	0.331 ±0.024	0.124 ±0.087	0.351 ±0.185	0.241 ±0.027	N.D.	N.D.	N.D.	N.D.	0.133 ±0.047	N.D.	A
41	872		Ethyl acetate	0.068 ±0.017	0.255 ±0.037	0.373 ±0.396	0.09 ±0.038	0.076 ±0.006	0.645 ±0.12	0.149 ±0.027	0.117 ±0.01	0.475 ±0.461	0.227 ±0.147	0.401 ±0.158	0.048 ±0.022	0.03 ±0.006	0.047 ±0.017	0.1 ±0.088	0.116 ±0.046	0.041 ±0.006	A
42	1174		Butyl prop-2-enoate	N.D.	0.074 ±0.02	N.D.	N.D.	N.D.	N.D.	N.D.	N.D.	N.D.	0.133 ±0.053	N.D.	1.052 ±1.003	N.D.	N.D.	0.951 ±0.394	N.D.	N.D.	A
43	1220		Butyl butanoate	N.D.	N.D.	N.D.	N.D.	N.D.	N.D.	N.D.	N.D.	N.D.	N.D.	N.D.	0.232 ±0.113	N.D.	N.D.	0.196 ±0.182	N.D.	N.D.	A
			**Furans**																	
44	775		Furan	N.D.	N.D.	N.D.	N.D.	N.D.	N.D.	N.D.	N.D.	N.D.	0.014 ±0.002	N.D.	N.D.	0.006 ±0.001	0.003 ±0.003	N.D.	N.D.	N.D.	A
45	850		2-Methylfuran	0.038 ±0.005	0.049 ±0.011	0.036 ±0.01	0.065 ±0.011	0.034 ±0.001	0.03 ±0.009	0.09 ±0.009	0.045 ±0.001	0.042 ±0.011	0.106 ±0.029	0.024 ±0.007	0.02 ±0.008	0.015 ±0.003	0.017 ±0.004	0.021 ±0.020	0.061 ±0.017	0.037 ±0.008	A
46	861		2,5-Dihydrofuran	N.D.	N.D.	N.D.	N.D.	N.D.	N.D.	N.D.	N.D.	N.D.	N.D.	N.D.	N.D.	0.005 ±0.001	0.009 ±0.004	0.058 ±0.100	N.D.	N.D.	A
47	826	832	3-Methylfuran	N.D.	N.D.	0.004 ±0.004	N.D.	N.D.	N.D.	0.033 ±0.003	0.007 ±0.000	0.004 ±0.003	N.D.	N.D.	N.D.	N.D.	N.D.	0.133 ±0.23	0.042 ±0.009	N.D.	B
48	940		2-Ethylfuran	0.137 ±0.019	0.242 ±0.095	0.117 ±0.003	0.297 ±0.092	0.037 ±0.005	0.069 ±0.002	0.319 ±0.047	0.178 ±0.018	0.167 ±0.073	0.515 ±0.189	0.063 ±0.007	0.074 ±0.024	0.069 ±0.01	0.126 ±0.068	0.1 ±0.028	0.353 ±0.075	0.182 ±0.047	A
49	1230		2-Pentylfuran	0.044 ±0.004	0.129 ±0.146	0.035 ±0.015	0.169 ±0.167	N.D.	N.D.	N.D.	0.045 ±0.009	0.083 ±0.014	0.072 ±0.028	N.D.	0.092 ±0.028	0.099 ±0.039	0.328 ±0.440	0.037 ±0.038	0.036 ±0.009	0.264 ±0.145	A
			**Ketones**																	
50	792		Propan-2-one	2.116 ±0.224	3.387 ±0.565	3.193 ±0.12	2.845 ±0.275	2.991 ±0.548	2.127 ±0.256	8.92 ±0.924	6.879 ±0.647	5.325 ±0.94	9.086 ±4.373	2.599 ±0.214	1.207 ±0.318	0.892 ±0.07	1.081 ±0.459	1.729 ±0.419	5.909 ±0.938	3.266 ±0.965	A
51	889		Butan-2-one	0.308 ±0.04	0.608 ±0.041	0.534 ±0.078	0.571 ±0.058	0.13 ±0.03	0.269 ±0.022	1.268± 0.117	0.539 ±0.474	0.514 ±0.05	1.019 ±0.321	0.405 ±0.092	0.133 ±0.029	0.289 ±0.029	0.314 ±0.163	16.19 ±27.353	1.02 ±0.24	0.453 ±0.063	A
52	966		Pentan-2-one	N.D.	N.D.	0.042 ±0.004	N.D.	N.D.	N.D.	0.312 ±0.038	0.133 ±0.009	N.D.	0.602 ±0.317	0.026 ±0.004	N.D.	N.D.	N.D.	N.D.	0.227 ±0.061	N.D.	A
53	1120	1121	(*E*)-Pent-3-en-2-one	N.D.	0.073 ±0.03	0.023 ±0.009	N.D.	N.D.	N.D.	N.D.	0.012 ±0.011	0.4 ±0.093	0.102 ±0.029	0.02 ±0.005	N.D.	N.D.	N.D.	N.D.	N.D.	N.D.	B
54	1178		Heptan-2-one	N.D.	0.026 ±0.023	N.D.	0.038 ±0.033	N.D.	N.D.	N.D.	N.D.	N.D.	N.D.	N.D.	N.D.	N.D.	N.D.	N.D.	N.D.	N.D.	A
55	1279		3-Hydroxybutan-2-one	0.029 ±0.006	N.D.	N.D.	N.D.	N.D.	N.D.	N.D.	N.D.	N.D.	N.D.	N.D.	N.D.	N.D.	N.D.	N.D.	N.D.	N.D.	A
56	1297		Oct-1-en-3-one	N.D.	N.D.	N.D.	N.D.	N.D.	N.D.	0.051 ±0.012	N.D.	N.D.	N.D.	N.D.	N.D.	N.D.	N.D.	N.D.	N.D.	N.D.	A
57	1336		6-Methylhept-5-en-2-one	N.D.	N.D.	0.05 ±0.009	N.D.	N.D.	0.034 ±0.008	0.042 ±0.014	N.D.	N.D.	N.D.	N.D.	0.102 ±0.031	0.043 ±0.011	0.044 ±0.012	0.045 ±0.014	N.D.	0.442 ±0.045	A
58	1490	1495	2-Decanone	N.D.	N.D.	N.D.	N.D.	N.D.	N.D.	N.D.	N.D.	N.D.	N.D.	N.D.	N.D.	N.D.	N.D.	N.D.	0.153 ±0.061	N.D.	B
59	1641	1645	1-Phenylethanone	N.D.	N.D.	N.D.	N.D.	N.D.	N.D.	N.D.	N.D.	N.D.	N.D.	N.D.	0.339 ±0.078	0.416 ±0.039	0.653 ±0.196	N.D.	N.D.	2.84 ±0.102	B
			**Lactones**																	
60	1600		5-Methyloxolan-2-one	N.D.	N.D.	N.D.	N.D.	N.D.	N.D.	0.071 ±0.014	N.D.	N.D.	N.D.	N.D.	N.D.	N.D.	N.D.	N.D.	N.D.	N.D.	A

61	1613		Oxolan-2-one	0.22 ±0.012	0.387 ±0.073	0.437 ±0.603	0.146 ±0.02	0.114 ±0.003	0.145 ±0.015	0.215 ±0.171	0.161 ±0.008	0.09 ±0.04	0.589 ±0.396	0.177 ±0.018	0.124 ±0.027	0.429 ±0.13	0.272 ±0.047	0.317 ±0.063	0.196 ±0.027	0.13 ±0.095	A
62	1688	1694	5-Ethyloxolan-2-one	N.D.	0.071 ±0.011	0.065 ±0.002	0.098 ±0.024	N.D.	N.D.	0.056 ±0.004	0.075 ±0.006	N.D.	0.137 ±0.049	0.074 ±0.004	0.046 ±0.007	0.041 ±0.004	0.074 ±0.022	0.079 ±0.020	N.D.	0.189 ±0.017	B
			**N-containing compounds**																		
63	<600		N,N-Dimethylmethanamine	N.D.	N.D.	N.D.	N.D.	N.D.	N.D.	N.D.	N.D.	N.D.	N.D.	N.D.	0.024 ±0.005	N.D.	0.038 ±0.029	0.028 ±0.010	N.D.	0.157 ±0.012	A
64	1178		Pyridine	N.D.	N.D.	N.D.	N.D.	N.D.	N.D.	N.D.	N.D.	N.D.	N.D.	0.03 ±0.005	N.D.	N.D.	0.055 ±0.051	N.D.	N.D.	N.D.	A
65	1782		Methyl (*Z*)-N-hydroxybenzenecarboximidate	0.058 ±0.011	N.D.	N.D.	0.054 ±0.027	0.048 ±0.015	N.D.	N.D.	N.D.	N.D.	N.D.	N.D.	0.095 ±0.029	0.071 ±0.017	0.073 ±0.052	N.D.	N.D.	N.D.	C
			**S-containing compounds**																		
66	716		Methylsulfanylmethane	0.114 ±0.073	0.121 ±0.01	0.083 ±0.015	0.076 ±0.039	0.006 ±0.002	0.029 ±0.003	0.621 ±0.123	0.423 ±0.08	0.053 ±0.045	0.566 ±0.078	0.039 ±0.003	0.033 ±0.023	0.198 ±0.223	0.127 ±0.041	0.221 ±0.048	0.64 ±0.197	0.307 ±0.069	A
67	1937		1,3-Benzothiazole	0.169 ±0.065	0.203 ±0.102	0.14 ±0.048	0.163 ±0.05	0.167 ±0.011	0.156 ±0.014	0.157 ±0.043	0.157 ±0.006	0.136 ±0.062	0.232 ±0.073	0.114 ±0.027	0.415 ±0.079	0.256 ±0.09	0.683 ±0.239	0.388 ±0.106	0.159 ±0.042	0.409 ±0.219	A
			**Hydrocarbons**																		
68	<600		2-Methylprop-1-ene	0.022 ±0.002	0.035 ±0.007	0.025 ±0.005	0.017 ±0.005	0.022 ±0.001	0.044 ±0.012	0.031 ±0.004	0.022 ±0.001	0.029 ±0.006	0.026 ±0.006	0.043 ±0.007	0.045 ±0.008	0.045 ±0.005	0.043 ±0.021	0.036 ±0.005	0.029 ±0.003	0.036 ±0.004	C
69	<600		Pentane	N.D.	0.026 ±0.019	0.025 ±0.004	N.D.	0.006 ±0.001	0.011 ±0.001	0.02 ±0.004	0.014 ±0.000	N.D.	0.225 ±0.36	0.011 ±0.000	N.D.	N.D.	N.D.	N.D.	0.015 ±0.001	0.011 ±0.006	A
70	600		Hexane	0.048 ±0.005	0.111 ±0.011	0.137 ±0.066	0.059 ±0.008	0.039 ±0.004	0.078 ±0.025	0.101 ±0.039	0.088 ±0.01	0.129 ±0.108	0.187 ±0.019	0.101 ±0.016	0.205 ±0.052	N.D.	N.D.	0.157 ±0.046	0.192 ±0.117	N.D.	A
71	613		1,1-Dichloro-1-fluoroethane	N.D.	N.D.	N.D.	N.D.	N.D.	N.D.	N.D.	N.D.	0.006 ±0.01	N.D.	N.D.	0.011 ±0.008	N.D.	N.D.	N.D.	0.022 ±0.024	N.D.	C
72	700		Heptane	N.D.	0.031 ±0.007	0.044 ±0.024	0.033 ±0.002	0.022 ±0.003	0.028 ±0.004	0.044 ±0.008	0.027 ±0.003	0.03 ±0.006	0.05 ±0.005	0.023 ±0.001	0.022 ±0.007	0.023 ±0.002	0.036 ±0.018	0.022 ±0.005	0.04 ±0.003	0.035 ±0.005	A
73	735	725	Methylcyclohexane	N.D.	N.D.	N.D.	N.D.	N.D.	N.D.	N.D.	N.D.	N.D.	N.D.	N.D.	0.002 ±0.004	0.013 ±0.005	0.017 ±0.001	N.D.	N.D.	0.015 ±0.004	B
74	800		Octane	0.025 ±0.004	0.03 ±0.005	0.043 ±0.046	0.045 ±0.007	N.D.	0.013 ±0.002	0.037 ±0.003	0.024 ±0.003	0.023 ±0.011	0.083 ±0.029	0.012 ±0.001	0.028 ±0.011	0.021 ±0.003	0.043 ±0.033	0.031 ±0.01	0.068 ±0.028	0.056 ±0.006	A
75	1198		Dodecane	0.05 ±0.087	N.D.	N.D.	N.D.	N.D.	N.D.	N.D.	N.D.	N.D.	N.D.	N.D.	0.256 ±0.135	0.108 ±0.027	0.24 ±0.041	0.17 ±0.082	N.D.	0.161 ±0.015	A
76	1277		Pentylcyclopentane	N.D.	N.D.	N.D.	N.D.	N.D.	N.D.	N.D.	N.D.	N.D.	N.D.	N.D.	0.022 ±0.037	N.D.	N.D.	0.178 ±0.118	N.D.	N.D.	C
77	1399	1400	Tetradecane	N.D.	N.D.	N.D.	N.D.	N.D.	N.D.	N.D.	N.D.	0.042 ±0.058	N.D.	N.D.	0.111 ±0.073	0.072 ±0.012	0.094 ±0.038	0.057 ±0.002	N.D.	0.107 ±0.008	B
			**Terpenes**																		
78	1092		β-Pinene	N.D.	N.D.	0.097 ±0.014	N.D.	N.D.	N.D.	N.D.	N.D.	N.D.	N.D.	N.D.	N.D.	N.D.	N.D.	N.D.	N.D.	N.D.	A
79	1157		l-Phellandrene	0.087 ±0.039	N.D.	N.D.	N.D.	N.D.	N.D.	N.D.	N.D.	N.D.	N.D.	N.D.	N.D.	N.D.	N.D.	N.D.	N.D.	N.D.	A
80	1190		Limonene	0.039 ±0.011	N.D.	0.028 ±0.003	N.D.	N.D.	N.D.	N.D.	N.D.	N.D.	0.196 ±0.132	N.D.	0.43 ±0.519	N.D.	0.134 ±0.231	0.127 ±0.083	N.D.	0.065 ±0.022	A
81	1198		Sabinene	0.142	N.D.	N.D.	N.D.	N.D.	N.D.	N.D.	N.D.	N.D.	N.D.	N.D.	0.042	N.D.	0.022	N.D.	N.D.	N.D.	A

				±0.143											±0.072		±0.038				
82	1243		γ-Terpinene	N.D.	N.D.	N.D.	N.D.	N.D.	N.D.	N.D.	N.D.	N.D.	0.5 ±0.723	N.D.	N.D.	N.D.	N.D.	N.D.	N.D.	N.D.	A
83	1736	1736	Azulene	N.D.	N.D.	N.D.	N.D.	N.D.	N.D.	N.D.	N.D.	N.D.	N.D.	N.D.	N.D.	N.D.	N.D.	N.D.	N.D.	0.109 ±0.019	B
			**Phenols**																		
84	1956		Phenol	N.D.	N.D.	N.D.	0.004 ±0.006	0.037 ±0.003	0.014 ±0.003	N.D.	N.D.	N.D.	N.D.	N.D.	0.031 ±0.01	0.02 ±0.005	0.025 ±0.025	N.D.	N.D.	0.034 ±0.001	A

[1] All volatile metabolites are listed by the order of their RI values; [2] Retention indices were determined using n-alkanes C_6 to C_{30} as an external standard; [3] Retention indices were obtained from NIST database(http://webbook.nist.gov/chemistry); [4] Mean values of relative peak area to that of internal standard ± standard deviation; [5] Not detected; [6] Identification of the compounds was based as follows; A, mass spectrum and retention index agree with the authentic compounds under similar conditions (positive identification); B, mass spectrum and retention index were consistent with those from NIST database; C, mass spectrum was consistent with that of W9N08 (Wiley and NIST) and manual interpretation (tentative identification); [7] Abbreviation are defined as shown in Table 4.

Table S2: Volatile metabolites of soybeans cultivated in China

No. [1]	RI cal [2]	RI ref [3]	Volatile Compounds	Relative Peak Area [4]													ID [6]
				INUL [7]	HEHA	JIME	LIDA	HESH	SHJI	HUHU	ANHU	ZHPI	FULO	JIJI	GUSH	GUBA	
			**Acids**														
1	1448		Acetic acid	2.803 ±0.023	N.D. [5]	N.D.	N.D.	0.033 ±0.014	0.021 ±0.013	N.D.	0.035 ±0.012	2.07 ±0.048	9.045 ±3.215	N.D.	7.543 ±1.880	2.55 ±0.266	A
2	1861	1854	Hexanoic acid	N.D.	N.D.	N.D.	N.D.	N.D.	N.D.	N.D.	N.D.	N.D.	1.355 ±0.489	N.D.	N.D.	N.D.	B
			**Alcohols**														
3	925		Propan-2-ol	N.D.	N.D.	N.D.	0.02 ±0.035	N.D.	N.D.	N.D.	N.D.	N.D.	N.D.	N.D.	N.D.	N.D.	A
4	930		Ethanol	4.229 ±1.103	0.86 ±0.098	4.304 ±1.362	0.96 ±0.033	9.618 ±3.136	3.465 ±0.890	4.822 ±5.155	25.695 ±3.938	8.165 ±0.833	20.333 ±11.479	3.426 ±0.827	4.168 ±0.999	2.998 ±0.478	A
5	1024		Butan-2-ol	0.278 ±0.033	0.148 ±0.009	0.253 ±0.151	0.084 ±0.006	0.346 ±0.178	0.162 ±0.035	0.159 ±0.189	0.275 ±0.044	0.269 ±0.029	0.755 ±0.305	0.399 ±0.104	0.626 ±0.303	0.259 ±0.049	A
6	1098		2-Methylpropan-1-ol	0.158 ±0.012	N.D.	N.D.	N.D.	N.D.	N.D.	N.D.	N.D.	0.27 ±0.101	0.598 ±0.560	N.D.	N.D.	N.D.	A
7	1129		1-Methoxypropan-2-ol	0.349 ±0.034	0.321 ±0.027	0.357 ±0.135	0.147 ±0.018	0.611 ±0.114	0.371 ±0.036	0.646 ±0.379	0.772 ±0.017	1.612 ±0.165	4.209 ±0.863	2.695 ±0.637	2.996 ±0.366	4.071 ±0.463	A
8	1151		Butan-1-ol	1.867 ±0.267	3.356 ±0.095	4.218 ±2.108	2.114 ±0.268	7.326 ±2.199	5.08 ±0.928	16.827 ±12.654	13.653 ±0.435	19.744 ±1.306	22.487 ±6.99	14.498 ±3.180	13.835 ±3.352	10.915 ±1.360	A
9	1194		2,5-Dimethylhexan-2-ol	N.D.	N.D.	N.D.	0.334 ±0.029	N.D.	N.D.	N.D.	N.D.	N.D.	0.51 ±0.140	N.D.	0.231 ±0.013	N.D.	C
10	1212		3-Methylbutan-1-ol	2.162 ±0.051	N.D.	N.D.	N.D.	N.D.	N.D.	N.D.	N.D.	1.732 ±0.955	2.792 ±1.953	N.D.	N.D.	N.D.	A
11	1255		Pentan-1-ol	0.356 ±0.005	0.159 ±0.021	0.342 ±0.066	0.348 ±0.043	0.347 ±0.222	0.348 ±0.046	1.138 ±1.256	0.827 ±0.160	0.811 ±0.371	3.591 ±1.275	0.221 ±0.030	0.679 ±0.208	1.173 ±0.306	A
12	1289	1288	Heptan-4-ol	0.031 ±0.011	0.048 ±0.005	0.062±0.02	0.023 ±0.002	0.138 ±0.087	0.103 ±0.026	0.233 ±0.156	0.247 ±0.027	0.148 ±0.022	0.245 ±0.121	0.217 ±0.052	0.072 ±0.032	0.105 ±0.012	B
13	1345		3-Methylheptan-4-ol	N.D.	N.D.	N.D.	N.D.	N.D.	N.D.	N.D.	0.147 ±0.026	N.D.	N.D.	0.086 ±0.071	N.D.	0.069 ±0.008	A
14	1358		Hexan-1-ol	1.492 ±0.028	0.969 ±0.186	1.52 ±0.492	3.028 ±0.329	1.89 ±1.579	1.532 ±0.345	8.174 ±10.29	4.77 ±0.994	2.757 ±0.901	9.973 ±3.294	1.009 ±0.116	0.903 ±0.018	1.834 ±0.330	A
15	1398		Octan-3-ol	N.D.	N.D.	N.D.	N.D.	N.D.	N.D.	N.D.	N.D.	0.239 ±0.111	0.571 ±0.170	N.D.	N.D.	N.D.	A
16	1402		2-Butoxyethanol	0.136 ±0.007	N.D.	N.D.	N.D.	0.533 ±0.232	N.D.	N.D.	N.D.	0.041 ±0.004	N.D.	0.197 ±0.053	N.D.	N.D.	A
17	1428		3-Methoxy-3-methylbutan-1-ol	N.D.	N.D.	N.D.	N.D.	N.D.	N.D.	N.D.	N.D.	0.234 ±0.032	1.235 ±0.450	7.942 ±1.853	0.432 ±0.088	0.303 ±0.049	A
18	1448		Oct-1-en-3-ol	N.D.	N.D.	N.D.	0.123 ±0.035	N.D.	0.104 ±0.031	N.D.	0.184 ±0.106	N.D.	N.D.	N.D.	N.D.	N.D.	A
19	1454		Heptan-1-ol	N.D.	N.D.	N.D.	N.D.	N.D.	N.D.	N.D.	0.151 ±0.016	N.D.	N.D.	N.D.	N.D.	N.D.	A
20	1493		2-Ethylhexan-1-ol	1.899 ±0.203	1.605 ±0.608	1.862 ±0.228	0.529 ±0.028	3.59 ±2.246	2.29 ±0.443	4.065 ±1.931	4.641 ±0.438	3.341 ±0.213	4.323 ±1.250	3.787 ±0.732	0.871 ±0.377	2.303 ±0.562	A

21	1565		Octan-1-ol	N.D.	N.D.	0.11 ±0.038	0.205 ±0.028	0.234 ±0.124	N.D.	0.339 ±0.313	N.D.	N.D.	0.559 ±0.163	0.121 ±0.026	N.D.	N.D.	A
22	1582	1580	Butane-2,3-diol	0.074 ±0.004	N.D.	N.D.	N.D.	N.D.	N.D.	0.387 ±0.306	N.D.	N.D.	0.399 ±0.184	N.D.	N.D.	N.D.	B
23	1594		Propane-1,2-diol	N.D.	N.D.	N.D.	N.D.	N.D.	N.D.	N.D.	N.D.	N.D.	N.D.	0.612 ±0.267	N.D.	N.D.	A
24	1642	1631	5-Methyl-2-propan-2-ylcyclohexan-1-ol	0.185 ±0.006	0.167 ±0.025	N.D.	0.106 ±0.012	0.246 ±0.078	N.D.	0.2 ±0.115	0.229 ±0.038	0.213 ±0.016	0.418 ±0.150	0.175 ±0.018	0.689 ±0.061	0.248 ±0.034	B
25	1666		Nonan-1-ol	N.D.	N.D.	N.D.	0.221 ±0.035	0.285 ±0.245	N.D.	0.336 ±0.443	N.D.	N.D.	0.41 1±0.161	N.D.	N.D.	N.D.	A
26	1759		2-Phenylpropan-2-ol	N.D.	N.D.	N.D.	N.D.	0.041 ±0.019	N.D.	N.D.	N.D.	N.D.	N.D.	N.D.	N.D.	N.D.	A
27	1875		Phenylmethanol	0.27 ±0.009	N.D.	N.D.	N.D.	0.579 ±0.248	0.23 ±0.028	0.328 ±0.192	N.D.	N.D.	N.D.	N.D.	1.737 ±0.875	0.286 ±0.025	A
			**Aldehydes**														
28	605		Acetaldehyde	N.D.	N.D.	0.181 ±0.084	N.D.	0.189 ±0.061	0.09 ±0.023	0.229 ±0.191	0.495 ±0.043	0.161 ±0.059	N.D.	0.097 ±0.048	N.D.	0.054 ±0.016	A
29	857		Butanal	N.D.	0.016 ±0.002	N.D.	N.D.	N.D.	N.D.	0.639 ±0.824	0.091 ±0.006	0.143 ±0.054	N.D.	0.114 ±0.037	N.D.	0.019 ±0.005	A
30	863		2-Methylprop-2-enal	N.D.	0.011 ±0.006	N.D.	N.D.	N.D.	N.D.	N.D.	N.D.	N.D.	N.D.	N.D.	N.D.	0.026 ±0.000	A
31	1074		Hexanal	0.406 ±0.012	1.889 ±0.312	2.878 ±0.389	2.468 ±0.076	2.456 ±0.340	2.43 ±0.636	5.467 ±2.274	6.196 ±1.010	0.767 ±0.121	10.439 ±3.156	1.429 ±0.245	1.721 ±0.489	5.322 ±2.829	A
32	1181		Heptanal	N.D.	0.337 ±0.048	0.3 ±0.032	0.291 ±0.019	0.739 ±0.127	0.353 ±0.047	0.65 ±0.306	0.598 ±0.074	N.D.	1.021 ±0.330	0.417 ±0.094	N.D.	0.331 ±0.025	A
33	1210		(*E*)-Hex-2-enal	N.D.	0.511 ±0.103	0.4 ±0.131	N.D.	0.409 ±0.186	0.37 ±0.049	1.27 ±0.901	0.895 ±0.202	N.D.	N.D.	N.D.	N.D.	0.515 ±0.125	A
34	1285	1287	Octanal	0.15 ±0.015	0.472 ±0.070	0.349 ±0.065	0.426 ±0.045	0.861 ±0.088	0.372 ±0.100	0.614 ±0.190	0.485 ±0.070	0.093 ±0.002	1.032 ±0.154	0.662 ±0.134	0.223 ±0.063	0.397 ±0.060	B
35	1317		(*E*)-Hept-2-enal	N.D.	N.D.	0.069 ±0.033	N.D.	0.076 ±0.015	0.04 ±0.015	0.133 ±0.112	0.1 ±0.020	N.D.	N.D.	N.D.	N.D.	N.D.	A
36	1391		Nonanal	0.571 ±0.074	2.673 ±0.323	2.068 ±0.325	3.235 ±0.193	6.729 ±0.874	2.079 ±0.734	3.405 ±0.612	2.15 ±0.244	0.86 ±0.124	3.724 ±0.958	3.92 ±0.625	0.709 ±0.183	2.361 ±0.137	A
37	1420		(*E*)-Oct-2-enal	N.D.	N.D.	0.145 ±0.064	N.D.	0.164 ±0.065	N.D.	0.317 ±0.265	N.D.	N.D.	N.D.	N.D.	N.D.	N.D.	A
38	1495		Decanal	N.D.	N.D.	N.D.	0.212 ±0.019	N.D.	N.D.	N.D.	N.D.	N.D.	N.D.	N.D.	N.D.	N.D.	A
39	1514		Benzaldehyde	0.527 ±0.054	0.369 ±0.022	0.487 ±0.038	0.208 ±0.003	0.842 ±0.152	0.402 ±0.061	0.651 ±0.125	0.486 ±0.032	0.369 ±0.304	0.685 ±0.185	0.517 ±0.072	17.114 ±14.95	1.229 ±0.248	A
40	1532	1542	(*E*)-Non-2-enal	N.D.	0.167 ±0.017	N.D.	0.364 ±0.023	N.D.	N.D.	N.D.	N.D.	N.D.	N.D.	N.D.	N.D.	N.D.	B
			**Benzenes**														
41	1027		Toluene	17.019 ±2.87	5.657 ±0.743	2.736 ±0.872	2.259 ±0.017	10.099 ±10.187	2.709 ±1.22	13.237 ±19.586	5.453 ±1.022	5.707 ±1.842	N.D.	3.99 ±2.072	7.867 ±3.989	1.45 ±0.030	A
42	1119		Ethylbenzene	0.221 ±0.047	0.162 ±0.016	0.156 ±0.048	0.059 ±0.005	N.D.	0.145 ±0.074	0.784 ±1.239	0.187 ±0.014	0.208 ±0.069	0.336 ±0.226	0.325 ±0.196	0.195 ±0.199	N.D.	A
43	1126		1,4-Xylene	N.D.	0.091	0.064	0.03	0.129	0.07	0.265	0.072	0.053	N.D.	0.256	N.D.	N.D.	A

					±0.011	±0.025	±0.015	±0.186	±0.045	±0.432	±0.011	±0.018		±0.151			
44	1133		1,3-Xylene	0.358 ±0.079	0.202 ±0.022	0.249 ±0.099	0.14 ±0.011	0.569 ±0.591	0.231 ±0.138	0.702 ±1.032	0.17 ±0.022	0.232 ±0.078	0.812 ±0.523	0.564 ±0.335	0.37 ±0.317	0.077 ±0.003	A
45	1250		Styrene	N.D.	N.D.	0.106 ±0.017	N.D.	0.246 ±0.177	0.122 ±0.031	0.283 ±0.262	0.099 ±0.05	N.D.	0.758 ±0.334	0.571 ±0.296	0.219 ±0.143	0.077 ±0.02	A
46	1264		1-Methyl-3-propan-2-ylbenzene	0.016 ±0.004	N.D.	N.D.	N.D.	0.192 ±0.212	0.047 ±0.030	0.145 ±0.181	0.059 ±0.014	0.219 ±0.126	0.657 ±0.403	0.074 ±0.046	0.241 ±0.239	0.046 ±0.015	A
47	1273		1,2,3-Trimethylbenzene	0.033 ±0.009	0.034 ±0.011	0.039 ±0.017	0.02 ±0.008	0.186 ±0.177	0.055 ±0.028	0.145 ±0.180	0.058 ±0.010	0.046 ±0.015	0.272 ±0.219	0.109 ±0.057	0.109 ±0.129	N.D.	A
48	1820	1817	1-Methoxy-4-[(*E*)-prop-1-enyl]benzene	N.D.	N.D.	N.D.	N.D.	N.D.	N.D.	N.D.	N.D.	N.D.	N.D.	0.152 ±0.028	N.D.	N.D.	B
			**Esters**														
49	810		Methyl acetate	N.D.	N.D.	N.D.	N.D.	N.D.	N.D.	N.D.	N.D.	N.D.	0.611 ±0.225	0.179 ±0.044	0.408 ±0.103	0.23 ±0.031	A
50	873		Ethyl acetate	0.481 ±0.044	N.D.	0.114 ±0.048	N.D.	0.28 ±0.221	N.D.	N.D.	0.421 ±0.076	0.401 ±0.111	1.775 ±1.085	0.496 ±0.220	0.796 ±0.386	0.273 ±0.092	A
51	947		Ethyl propanoate	N.D.	N.D.	N.D.	N.D.	N.D.	N.D.	N.D.	0.031 ±0.003	N.D.	N.D.	N.D.	N.D.	N.D.	A
52	1067		Butyl acetate	4.851 ±1.253	0.373 ±0.035	2.314 ±0.372	0.438 ±0.103	6.813 ±6.189	1.439 ±0.818	6.744 ±8.587	2.022 ±0.543	0.475 ±0.047	5.451 ±3.763	5.333 ±2.749	4.264 ±1.760	2.507 ±0.340	A
53	1141		Butyl propanoate	1.965 ±0.636	0.931 ±0.035	2.016 ±0.229	0.545 ±0.103	4.842 ±4.519	1.73 ±0.902	6.1 ±7.201	3.112 ±0.451	0.36 ±0.141	4.284 ±2.817	3.599 1.821	1.438 ±1.303	1.306 ±0.155	A
54	1175		Butyl prop-2-enoate	1.139 ±0.325	1.022 ±0.080	1.127 ±0.189	0.467 ±0.054	2.585 ±2.434	1.182 ±0.461	3.213 ±3.434	1.832 ±0.225	0.516 ±0.078	3.469 ±2.101	1.91 ±0.887	1.35 ±1.185	0.661 ±0.05	A
55	1217		Butyl butanoate	3.191 ±1.332	3.425 ±0.271	4.822 ±0.995	1.452 ±0.208	10.555 ±8.953	5.871 ±3.489	13.69 ±12.426	12.291 ±1.299	1.681 ±0.327	15.808 ±9.685	8.919 ±4.203	3.205 ±3.700	3.145 ±0.311	A
56	1234		Ethyl hexanoate	N.D.	N.D.	0.059 ±0.053	N.D.	0.283 ±0.125	0.052 ±0.017	0.083 ±0.040	0.159 ±0.021	N.D.	0.359 ±0.150	N.D.	N.D.	0.082 ±0.018	A
57	1384		2-Ethylhexyl acetate	N.D.	N.D.	N.D.	N.D.	N.D.	N.D.	0.124 ±0.099	N.D.	N.D.	N.D.	N.D.	N.D.	N.D.	A
58	1479		2-Ethylhexyl prop-2-enoate	0.111 ±0.119	0.195 ±0.062	N.D.	N.D.	N.D.	N.D.	0.405 ±0.162	1.095 ±0.309	N.D.	N.D.	N.D.	N.D.	N.D.	A
59	1664	1672	Triethyl phosphate	N.D.	N.D.	N.D.	N.D.	N.D.	N.D.	N.D.	1.992 ±1.250	N.D.	N.D.	N.D.	N.D.	N.D.	B
60	1765		Methyl 2-hydroxybenzoate	N.D.	N.D.	N.D.	N.D.	N.D.	N.D.	N.D.	0.486 ±0.068	N.D.	N.D.	N.D.	0.39 ±0.131	N.D.	A
61	1850		3-Hydroxy-2,4,4-trimethylpentyl 2-methylpropanoate	0.06 ±0.004	0.399 ±0.245	0.218 ±0.202	0.235 ±0.099	N.D.	N.D.	N.D.	N.D.	N.D.	N.D.	N.D.	0.446 ±0.153	N.D.	C
			**Furans**														
62	863	851	3-Methylfuran	N.D.	N.D.	N.D.	0.035 ±0.014	0.01 ±0.001	N.D.	N.D.	0.033 ±0.010	0.019 ±0.003	0.183 ±0.069	0.021 ±0.003	N.D.	0.013 ±0.002	B
63	873		2-Methylfuran	0.039 ±0.004	0.009 ±0.005	0.013 ±0.004	0.004 ±0.001	N.D.	0.018 ±0.002	0.05 ±0.064	0.02 ±0.007	0.02 ±0.004	0.055 ±0.021	0.018 ±0.008	N.D.	0.076 ±0.001	A
64	941		2-Ethylfuran	0.088 ±0.014	0.116 ±0.034	0.142 ±0.040	0.181 ±0.015	0.064 ±0.014	0.154 ±0.047	0.793 ±1.026	0.38 ±0.084	0.113 ±0.041	0.742 ±0.306	0.113 ±0.024	0.213 ±0.099	0.2 ±0.033	A
65	1230		2-Pentylfuran	0.057	0.104	0.314	0.074	0.369	0.334	0.745	0.357	N.D.	0.57	0.079	0.139	0.155	A

				±0.004	±0.019	±0.140	±0.015	±0.139	±0.015	±0.534	±0.103		±0.163	±0.011	±0.008	±0.057	
			**Ketones**														
66	805		Propan-2-one	3.145 ±0.203	N.D.	1.938 ±0.338	1.551 ±0.035	1.318 ±0.910	0.939 ±0.292	6.598 ±8.154	3.108 ±0.657	2.176 ±0.256	5.92 ±2.770	1.356 ±0.203	3.481 ±0.914	6.734 ±1.240	A
67	890		Butan-2-one	0.35 ±0.032	0.221 ±0.046	0.379 ±0.043	0.428 ±0.043	0.242 ±0.116	0.328 ±0.071	1.181 ±1.375	0.733 ±0.154	1.005 ±0.177	0.908 ±0.39	0.451 ±0.072	0.692 ±0.302	0.848 ±0.127	A
68	944		Bicyclo[4.1.0]heptan-5-one	N.D.	N.D.	N.D.	N.D.	N.D.	N.D.	N.D.	N.D.	0.018 ±.0180	N.D.	N.D.	N.D.	N.D.	C
69	966		Pentan-2-one	0.081 ±0.011	N.D.	0.038 ±0.006	0.155 ±0.021	0.03 ±0.017	0.057 ±0.015	N.D.	N.D.	0.834 ±0.376	2.211 ±0.911	0.034 ±0.014	0.141 ±0.026	0.84 ±0.133	A
70	1097		1-Methoxypropan-2-one	N.D.	N.D.	N.D.	N.D.	N.D.	N.D.	N.D.	N.D.	N.D.	N.D.	N.D.	N.D.	0.28 ±0.046	A
71	1121	1121	(*E*)-Pent-3-en-2-one	N.D.	N.D.	N.D.	N.D.	1.221 ±0.294	N.D.	N.D.	N.D.	N.D.	1.591 ±0.524	N.D.	N.D.	2.298 ±0.303	B
72	1122		Heptan-4-one	N.D.	0.167 ±0.028	0.148 ±0.003	N.D.	N.D.	0.194 ±0.048	0.473 ±0.387	0.419 ±0.046	0.33 ±0.004	N.D.	0.291 ±0.081	N.D.	N.D.	A
73	1145		3-Methylheptan-4-one	0.187 ±0.077	0.499 ±0.032	0.411 ±0.052	0.177 ±0.020	0.909 ±0.850	0.596 ±0.239	1.855 ±2.112	1.119 ±0.115	0.789 ±0.234	2.281 ±1.624	0.962 ±0.515	1.074 ±1.047	0.254 ±0.024	C
74	1171		2,6-Dimethylheptan-4-one	N.D.	N.D.	N.D.	N.D.	N.D.	N.D.	1.98 ±3.064	N.D.	N.D.	N.D.	N.D.	N.D.	N.D.	A
75	1178		Heptan-2-one	N.D.	N.D.	0.049 ±0.005	0.065 ±0.004	0.025 ±0.019	0.063 ±0.008	0.17 ±0.155	0.107 ±0.020	0.091 ±0.019	0.348 ±0.136	N.D.	0.06 ±0.007	0.118 ±0.065	A
76	1252		Octan-3-one	N.D.	N.D.	N.D.	N.D.	N.D.	N.D.	N.D.	N.D.	0.246 ±0.108	N.D.	N.D.	N.D.	N.D.	A
77	1279		3-Hydroxybutan-2-one	N.D.	N.D.	N.D.	N.D.	N.D.	N.D.	0.182 ±0.136	N.D.	0.074 ±0.027	0.182 ±0.073	N.D.	N.D.	0.122 ±0.034	A
78	1282		Octan-2-one	N.D.	N.D.	N.D.	0.088 ±0.010	N.D.	N.D.	N.D.	N.D.	0.068 ±0.026	N.D.	N.D.	N.D.	N.D.	A
79	1335		6-Methylhept-5-en-2-one	0.099 ±0.019	0.097 ±0.014	0.153 ±0.098	0.086 ±0.010	0.271 ±0.040	0.092 ±0.013	N.D.	0.106 ±0.017	0.086 ±0.006	N.D.	0.085 ±0.01	0.063 ±0.03	0.1 ±0.032	A
80	1402	1414	(*E*)-Oct-3-en-2-one	N.D.	N.D.	N.D.	N.D.	N.D.	N.D.	N.D.	N.D.	N.D.	0.531 ±0.219	N.D.	N.D.	0.184 ±0.03	B
81	1489	1495	2-Decanone	N.D.	N.D.	N.D.	N.D.	N.D.	0.162 ±0.040	N.D.	N.D.	N.D.	N.D.	N.D.	N.D.	N.D.	B
82	1584		3-Methylcyclohex-2-en-1-one	N.D.	N.D.	N.D.	N.D.	N.D.	N.D.	N.D.	N.D.	N.D.	0.171 ±0.114	N.D.	N.D.	N.D.	A
			**Lactones**														
83	1596		5-Methyloxolan-2-one	N.D.	N.D.	N.D.	N.D.	N.D.	N.D.	N.D.	N.D.	0.21 ±0.007	0.255 ±0.103	N.D.	0.109 ±0.018	N.D.	A
84	1613		Oxolan-2-one	0.455 ±0.006	N.D.	0.163 ±0.023	0.154 ±0.029	0.228 ±0.092	N.D.	0.642 ±0.838	0.266 ±0.013	0.687 ±0.219	1.375 ±0.670	0.343 ±0.066	0.258 ±0.014	0.221 ±0.022	A
85	1688	1694	5-Ethyloxolan-2-one	0.115 ±0.002	0.146 ±0.002	0.244 ±0.029	0.151 ±0.019	0.149 ±0.078	0.152 ±0.013	0.585 ±0.458	0.339 ±0.043	0.162 ±0.021	0.352 ±0.165	N.D.	0.14 0±0.015	0.169 ±0.032	B
86	1991	2005	5-Pentyloxolan-2-one	N.D.	N.D.	N.D.	N.D.	N.D.	N.D.	N.D.	N.D.	N.D.	0.183 ±0.081	N.D.	N.D.	N.D.	B
			**N-containing compounds**														

87	<600		N,N-Dimethylmethanamine	0.065 ±0.045	0.056 ±0.018	N.D.	0.064 ±0.012	0.032 ±0.011	N.D.	N.D.	0.061 ±0.015	N.D.	0.244 ±0.228	0.081 ±0.039	0.05 ±0.038	0.012 ±0.004	A
88	995		Acetonitrile	N.D.	N.D.	N.D.	N.D.	N.D.	N.D.	N.D.	N.D.	1.392 ±0.102	3.495 ±1.868	2.29 ±0.640	3.031 ±1.475	2.048 ±0.675	A
89	1725		Naphthalene	N.D.	0.097 ±0.004	0.084 ±0.038	N.D.	0.197 ±0.108	0.068 ±0.025	N.D.	N.D.	N.D.	N.D.	N.D.	N.D.	N.D.	A
			**S-containing compounds**														
90	<800		Methylsulfanylmethane	0.258 ±0.192	0.176 ±0.057	0.116 ±0.056	0.092 ±0.015	0.083 ±0.035	0.137 ±0.032	0.276 ±0.336	0.191 ±0.049	0.176 ±0.004	0.905 ±0.628	0.044 ±0.006	0.081 ±0.006	0.291 ±0.100	A
91	1937		1,3-Benzothiazole	0.29 ±0.135	0.611 ±0.09	0.182 ±0.034	0.359 ±0.327	0.599 ±0.267	0.348 ±0.084	0.395 ±0.120	0.202 ±0.059	0.277 ±0.115	0.571 ±0.770	0.28 ±0.080	0.285 ±0.013	0.276 ±0.030	A
			**Hydrocarbons**														
92	<600		2-Methylprop-1-ene	N.D.	0.043 ±0.003	0.047 ±0.011	0.04 ±0.003	0.076 ±0.026	0.01 5±0.006	0.059 ±0.025	0.033 ±0.009	0.033 ±0.005	0.029 ±0.004	0.026 ±0.006	0.021 ±0.003	0.019 ±0.006	C
93	<600		Pentane	N.D.	N.D.	0.003 ±0.003	N.D.	N.D.	N.D.	0.023 ±0.006	N.D.	0.046 ±0.006	0.052 ±.052	N.D.	N.D.	N.D.	A
94	600		Hexane	N.D.	0.064 ±0.005	0.051 ±0.012	0.038 ±0.006	0.037 ±0.019	0.028 ±0.008	0.097 ±0.129	0.08 ±0.036	0.115 ±0.023	0.205 ±0.075	0.115 ±0.041	0.139 ±0.056	0.082 ±0.039	A
95	700		Heptane	0.039 ±0.004	N.D.	0.053 ±0.037	0.032 ±0.002	0.057 ±0.036	0.05 ±0.024	0.139 ±0.201	0.044 ±0.004	0.057 ±0.013	0.386 ±0.165	0.046 ±0.014	0.081 0.024	0.046 ±0.004	A
96	763		2-tert-Butylperoxy-2-methylpropane	0.267 ±0.100	0.088 ±0.046	0.151 ±0.07	0.063 ±0.041	1.31 ±1.858	N.D.	2.404 ±4.060	0.143 ±0.069	0.787 ±0.464	2.789 ±1.511	0.566 ±0.557	1.554 ±0.925	0.114 ±0.016	C
97	800		Octane	0.17 ±0.050	N.D.	0.056 ±0.017	0.075 ±0.012	N.D.	N.D.	N.D.	0.083 ±0.011	N.D.	N.D.	0.102 ±0.024	0.428 ±0.103	0.086 ±0.009	A
98	875		Ethylcyclohexane	N.D.	N.D.	N.D.	N.D.	N.D.	0.516 ±0.354	N.D.	N.D.	N.D.	N.D.	N.D.	N.D.	N.D.	C
99	959	968	1-Butoxybutane	1.163 ±0.527	0.702 ±0.065	0.808 ±0.324	0.259 ±0.059	2.88 ±3.553	0.934 ±0.715	5.776 ±9.205	0.035 ±0.016	1.631 ±0.812	6.569 ±4.542	1.576 ±1.165	3.233 ±2.813	0.355 ±0.024	B
100	999		Decane	0.121 ±0.020	N.D.	N.D.	0.026 ±0.010	0.139 ±0.176	0.241 ±0.229	N.D.	N.D.	N.D.	N.D.	N.D.	0.47 ±0.429	N.D.	A
101	1093	1100	Undecane	N.D.	N.D.	N.D.	N.D.	N.D.	N.D.	1.506 ±2.508	N.D.	N.D.	N.D.	N.D.	N.D.	N.D.	B
102	1198		Dodecane	0.159 ±0.031	0.108 ±0.014	0.171 ±0.079	0.115 ±0.009	0.632 ±0.542	0.312 ±0.203	1.131 ±1.628	0.164 ±0.038	N.D.	N.D.	0.383 ±0.143	0.443 ±0.384	0.179 ±0.063	A
103	1299	1300	Tridecane	N.D.	N.D.	N.D.	N.D.	0.545 ±0.3	N.D.	0.22 ±0.232	N.D.	N.D.	0.175 ±0.086	N.D.	N.D.	N.D.	B
104	1399	1400	Tetradecane	0.089 ±0.005	0.156 ±0.016	0.119 ±0.062	0.133 ±0.014	0.162 ±0.067	0.15 ±0.025	0.159 ±0.091	N.D.	N.D.	N.D.	0.209 ±0.057	N.D.	0.062 ±0.015	B
105	1456		1-Chloro-2-(1-chloropropan-2-yloxy)propane	N.D.	N.D.	N.D.	N.D.	N.D.	N.D.	7.688 ±9.274	N.D.	N.D.	N.D.	N.D.	N.D.	N.D.	C
106	1460		1-Chloro-3-(3-chloropropoxy)propane	N.D.	N.D.	N.D.	N.D.	N.D.	N.D.	6.413 ±8.046	N.D.	N.D.	N.D.	N.D.	N.D.	N.D.	C
107	1466		2-Chloro-2-(2-chloropropan-2-yloxy)propane	N.D.	N.D.	N.D.	N.D.	N.D.	N.D.	7.518 ±8.897	N.D.	N.D.	N.D.	N.D.	N.D.	N.D.	C
			**Terpenes**														
108	1013		α-Pinene	N.D.	N.D.	N.D.	N.D.	N.D.	N.D.	N.D.	N.D.	N.D.	0.201	N.D.	0.29	N.D.	A

												±0.158		±0.188		
109	1018	1019	α-Thujene	N.D.	N.D.	N.D.	0.026 ±0.005	N.D.	N.D.	N.D.	N.D.	0.512 ±0.570	1.1 ±0.857	N.D.	0.191 ±0.169	N.D.	B
110	1112		Sabinene	N.D.	N.D.	N.D.	0.016 ±0.003	N.D.	N.D.	N.D.	N.D.	0.576 ±0.656	1.429 ±1.261	N.D.	0.245 ±0.262	N.D.	A
111	1157		l-Phellandrene	N.D.	N.D.	N.D.	N.D.	N.D.	N.D.	N.D.	N.D.	0.041 ±0.002	0.123 ±0.05	N.D.	N.D.	N.D.	A
112	1162		Myrcene	N.D.	N.D.	N.D.	N.D.	0.415 ±0.485	0.114 ±0.093	0.447 ±0.646	0.067 ±0.021	0.077 ±0.023	0.518 ±0.423	0.152 ±0.106	0.642 ±0.752	0.073 ±0.028	A
113	1172		α-Terpinene	N.D.	N.D.	N.D.	N.D.	N.D.	N.D.	N.D.	N.D.	0.187 ±0.108	0.642 ±0.455	N.D.	0.091 ±0.079	N.D.	A
114	1190		Limonene	0.212 ±0.042	0.139 ±0.026	0.31 ±0.096	0.095 ±0.018	1.967 ±2.177	0.464 ±0.371	1.7 ±2.451	0.471 ±0.11	0.675 ±0.193	3.23 ±2.326	0.783 ±0.520	2.149 ±2.310	0.319 ±0.124	A
115	1198	1196	β-Phellandrene	N.D.	N.D.	N.D.	N.D.	N.D.	N.D.	N.D.	N.D.	0.205 ±0.035	0.594 ±0.250	N.D.	N.D.	N.D.	B
116	1240		γ-Terpinene	N.D.	N.D.	N.D.	N.D.	0.138 ±0.130	0.04 ±0.029	N.D.	N.D.	0.316 ±0.200	1.131 ±0.792	0.041 ±0.027	0.252 ±0.247	0.031 ±0.014	A
117	1277		Terpinolene	N.D.	N.D.	N.D.	N.D.	N.D.	N.D.	N.D.	N.D.	0.065 ±0.031	0.24 ±0.169	N.D.	N.D.	N.D.	A
118	1559		α-Cedrene	N.D.	N.D.	N.D.	N.D.	N.D.	N.D.	N.D.	N.D.	N.D.	N.D.	N.D.	0.889 ±0.881	N.D.	A
			**Phenols**														
119	1956		Phenol	0.028 ±0.004	N.D.	0.028 ±0.003	N.D.	0.377 ±0.157	N.D.	N.D.	N.D.	N.D.	0.126 ±0.044	0.082 ±0.017	N.D.	N.D.	A
120	2054		4-Methylphenol	N.D.	N.D.	N.D.	N.D.	0.046 ±0.019	N.D.	N.D.	N.D.	N.D.	0.128 ±0.064	N.D.	N.D.	N.D.	A
121	2059		3-Methylphenol	N.D.	N.D.	N.D.	N.D.	0.096 ±0.037	N.D.	N.D.	N.D.	N.D.	N.D.	N.D.	N.D.	N.D.	A
			**Pyrazines**														
122	1261	1263	2-Methylpyrazine	0.096 ±0.001	N.D.	N.D.	N.D.	N.D.	N.D.	N.D.	N.D.	N.D.	N.D.	N.D.	N.D.	N.D.	B
123	1317		2,5-Dimethylpyrazine	0.066 ±0.003	N.D.	N.D.	N.D.	N.D.	N.D.	N.D.	N.D.	N.D.	N.D.	N.D.	N.D.	N.D.	A
124	1324		2,6-Dimethylpyrazine	0.07 ±0.006	N.D.	N.D.	N.D.	N.D.	N.D.	N.D.	N.D.	N.D.	N.D.	N.D.	N.D.	N.D.	A

[1] All volatile metabolites are listed by the order of their RI values; [2] Retention indices were determined using n-alkanes C_6 to C_{30} as an external standard; [3] Retention indices were obtained from NIST database(http://webbook.nist.gov/chemistry); [4] Mean values of relative peak area to that of internal standard ± standard deviation; [5] Not detected; [6] Identification of the compounds was based as follows; A, mass spectrum and retention index agree with the authentic compounds under similar conditions (positive identification); B, mass spectrum and retention index were consistent with those from NIST database; C, mass spectrum was consistent with that of W9N08 (Wiley and NIST) and manual interpretation (tentative identification); [7] Abbreviation are defined as shown in Table 4.

Table S3: Volatile metabolites of soybeans cultivated in North America

No. [1]	RI cal [2]	RI ref [3]	Volatile Compounds	Relative Peak Area [4]						ID [6]
				IL [7]	IN	MI	MN	ON	QB	
			**Acids**							
1	1445		Acetic acid	2.637±0.079	N.D. [5]	N.D.	2.389±0.695	5.617±1.504	N.D.	A
			**Alcohols**							
2	928		Ethanol	6.584±0.519	N.D.	N.D.	3.026±1.061	N.D.	N.D.	A
3	1022		Butan-2-ol	0.799±0.045	0.281±0.017	0.481±0.072	0.371±0.082	N.D.	0.364±0.080	A
4	1098		2-Methylpropan-1-ol	0.104±0.037	0.369±0.054	0.145±0.009	N.D.	N.D.	0.106±0.024	A
5	1128		1-Methoxypropan-2-ol	N.D.	N.D.	0.592±0.098	N.D.	N.D.	N.D.	A
6	1150		Butan-1-ol	N.D.	N.D.	N.D.	0.327±0.112	0.272±0.065	N.D.	A
7	1209		2-Methylbutan-1-ol	N.D.	3.793±0.454	N.D.	N.D.	N.D.	N.D.	C
8	1209		3-Methylbutan-1-ol	1.674±0.525	N.D.	N.D.	N.D.	2.890±1.063	N.D.	A
9	1253		Pentan-1-ol	0.759±0.176	0.124±0.010	0.175±0.063	0.067±0.029	0.571±0.168	N.D.	A
10	1355		Hexan-1-ol	2.837±0.601	0.432±0.086	1.014±0.567	N.D.	2.456±0.851	N.D.	A
11	1387		Octan-3-ol	N.D.	0.165±0.111	N.D.	N.D.	N.D.	N.D.	A
12	1400		2-Butoxyethanol	0.190±0.044	N.D.	N.D.	0.082±0.023	0.146±0.03	N.D.	A
13	1447		Oct-1-en-3-ol	2.929±0.280	7.134±0.957	4.160±0.65	N.D.	4.168±1.256	2.481±0.760	A
14	1492		2-Ethylhexan-1-ol	2.938±0.233	3.151±0.361	1.667±0.163	0.811±0.182	1.696±0.367	2.895±0.269	A
15	1587	1589	2-(2-Methoxyethoxy)ethanol	0.078±0.024	N.D.	N.D.	N.D.	N.D.	N.D.	B
16	1828		Phenylmethanol	N.D.	N.D.	N.D.	0.168±0.085	N.D.	N.D.	A
17	1920		2,2,4-Trimethylpentane-1,3-diol	N.D.	3.087±0.516	N.D.	N.D.	N.D.	N.D.	C
			**Aldehydes**							
18	1073		Hexanal	2.776±0.713	N.D.	0.932±0.164	0.837±0.463	1.566±0.509	0.259±0.133	A
19	1180		Heptanal	0.121±0.005	N.D.	N.D.	0.084±0.062	N.D.	N.D.	A
20	1282		Octanal	N.D.	N.D.	N.D.	0.134±0.157	0.104±0.013	N.D.	B
21	1389		Nonanal	1.110±0.179	N.D.	0.523±0.036	1.106±0.843	0.721±0.270	0.341±0.424	A
22	1513		Benzaldehyde	0.082±0.019	N.D.	N.D.	0.094±0.014	N.D.	N.D.	A
			**Benzenes**							
23	1025		Toluene	N.D.	N.D.	N.D.	0.721±0.533	N.D.	1.178±0.333	A
24	1118		Ethylbenzene	N.D.	0.079±0.012	N.D.	0.04±0.024	0.208±0.092	0.092±0.001	A
25	1131		1,2-Xylene	N.D.	0.082±0.032	0.135±0.043	0.047±0.032	0.349±0.143	0.082±0.006	A
26	1170		1,3-Xylene	0.078±0.022	N.D.	N.D.	N.D.	0.239±0.085	0.049±0.012	A
			**Esters**							
27	866		Ethyl acetate	N.D.	N.D.	N.D.	N.D.	0.039±0.015	N.D.	A
28	1140		Butyl propanoate	N.D.	N.D.	N.D.	0.083±0.048	N.D.	N.D.	A
29	1215		Butyl butanoate	N.D.	N.D.	N.D.	0.252±0.160	N.D.	N.D.	A
30	1850		3-Hydroxy-2,4,4-trimethylpentyl 2-methylpropanoate	N.D.	21.510±4.828	0.151±0.017	0.107±0.060	0.164±0.067	0.093±0.014	C
			**Furans**							
31	938		2-Ethylfuran	0.142±0.078	0.330±0.055	N.D.	0.279±0.08	0.395±0.042	0.085±0.005	A
32	1227		2-Pentylfuran	0.098±0.011	0.083±0.015	0.063±0.018	0.031±0.013	0.149±0.025	0.022±0.011	A
			**Ketones**							

33	886		Butan-2-one	N.D.	0.065±0.016	N.D.	N.D.	N.D.	N.D.	A
34	964		Pentan-2-one	N.D.	0.340±0.044	N.D.	N.D.	0.268±0.078	N.D.	A
35	1177		Heptan-2-one	N.D.	0.123±0.013	N.D.	N.D.	N.D.	N.D.	A
36	1249		Octan-3-one	0.088±0.055	2.153±0.083	0.140±0.064	N.D.	N.D.	0.456±0.177	A
			**Lactones**							
37	1598		4-Methyloxolan-2-one	0.270±0.028	N.D.	0.238±0.028	N.D.	0.631±0.133	N.D.	C
38	1612		Oxolan-2-one	0.468±0.052	0.086±0.05	N.D.	0.379±0.102	0.701±0.106	N.D.	A
39	1686	1694	5-Ethyloxolan-2-one	N.D.	N.D.	N.D.	0.137±0.039	N.D.	N.D.	B
			**N-containing compounds**							
40	<600		N,N-Dimethylmethanamine	N.D.	N.D.	N.D.	N.D.	0.023±0.005	N.D.	A
			**S-containing compounds**							
41	1937		1,3-Benzothiazole	0.244±0.138	0.322±0.112	0.2±0.041	0.101±0.01	0.295±0.143	0.093±0.072	A
			**Hydrocarbons**							
42	800		Octane	N.D.	0.077±0.024	N.D.	0.036±0.026	0.042±0.012	0.051±0.011	A
43	900	900	Nonane	N.D.	0.068±0.023	0.049±0.02	0.045±0.018	N.D.	0.042±0.008	B
44	1000		Decane	N.D.	0.073±0.077	N.D.	N.D.	0.158±0.088	0.094±0.000	A
45	1100		Dodecane	0.210±0.034	N.D.	0.172±0.024	0.092±0.038	0.185±0.068	N.D.	A
46	1397	1400	Tetradecane	N.D.	N.D.	0.167±0.093	0.194±0.145	N.D.	N.D.	B
			**Terpenes**							
47	1165		Cumene	N.D.	N.D.	N.D.	0.061±0.01	N.D.	N.D.	A
48	1188		Limonene	0.112±0.048	N.D.	0.095±0.059	N.D.	0.204±0.127	N.D.	A
			**Phenols**							
49	1956		Phenol	N.D.	N.D.	N.D.	0.046±0.012	N.D.	N.D.	A
			**Pyrazines**							
50	1258	1263	2-Methylpyrazine	N.D.	N.D.	N.D.	N.D.	0.063±0.011	N.D.	B

[1] All volatile metabolites are listed by the order of their RI values; [2] Retention indices were determined using n-alkanes C_6 to C_{30} as an external standard; [3] Retention indices were obtained from NIST database(http://webbook.nist.gov/chemistry); [4] Mean values of relative peak area to that of internal standard ± standard deviation; [5] Not detected; [6] Identification of the compounds was based as follows; A, mass spectrum and retention index agree with the authentic compounds under similar conditions (positive identification); B, mass spectrum and retention index were consistent with those from NIST database; C, mass spectrum was consistent with that of W9N08 (Wiley and NIST) and manual interpretation (tentative identification); [7] Abbreviation are defined as shown in Table 4.

Table S4: The provinces, cities, and geographic coordinates of soybean samples harvested in 2016 from Republic of Korea and China.

	No.	Province	City	Geographic coordinate
Korea	1	Gyeonggi	Anseong	N37°, E127°
	2	Gyeonggi	Icheon	N37°, E127°
	3	Gangwon	Chuncheon	N37°, E127°
	4	Gangwon	Yeongwol	N37°, E128°
	5	Chungcheongbuk	Eumseong	N36°, E127°
	6	Chungcheongnam	Cheonan	N36°, E127°
	7	Chungcheongnam	Gongju	N36°, E127°
	8	Jeollabuk	Gimje	N35°, E127°
	9	Jeollabuk	Imsil	N35°, E127°
	10	Jeollanam	Naju	N35°, E127°
	11	Jeollanam	Yeonggwang	N35°, E127°
	12	Kyeongsangbuk	Cheongdo	N36°, E128°
	13	Kyeongsangbuk	Uiseong	N36°, E128°
	14	Kyeongsangbuk	Yeongcheon	N36°, E128°
	15	Kyeongsangnam	Changnyeong	N35°, E128°
	16	Kyeongsangnam	Miryang	N35°, E128°
	17	Kyeongsangnam	Geochang	N35°, E127°
China	1	Neimenggu	Ulanhot	N40°, E111°
	2	Heilongjiang	Harbin	N45°, E126°
	3	Jilin	Meihekou	N42°, E125°
	4	Liaoning	Dandong	N40°, E124°
	5	Hebei	Shijiazhuang	N38°, E114°
	6	Shandong	Jining	N35°, E116°
	7	Anhui	Huaibei	N33°, E116°
	8	Hubei	Huangshi	N30°, E115°
	9	Zhejiang	Jiaxing	N30°, E120°
	10	Jiangxi	Jiujiang	N29°, E115°
	11	Fujian	Longyan	N25°, E117°
	12	Guangdong	Shaoguan	N24°, E113°
	13	Guangxi	Hechi	N24°, E108°
North America	1	Illinois		N39°, E-89°
	2	Indiana		N39°, E-86°
	3	Minnesota		N45°, E-94°
	4	Michigan		N43°, E-84°
	5	Quebec		N51°, E-72°
	6	Ontario		N51°, E-85°

*Korean soybean samples were provided by the National Agricultural Products Quality Management Service.
